# Transcutaneous electrical acupoint stimulation for children with attention-deficit/hyperactivity disorder: a randomized clinical trial

**DOI:** 10.1038/s41398-022-01914-0

**Published:** 2022-04-21

**Authors:** Lixia Zhuo, Xiaoyan Zhao, Yifang Zhai, Boqiang Zhao, Lin Tian, Yannan Zhang, Xiaodan Wang, Tingyu Zhang, Xinyi Gan, Cheng Yang, Weigang Wang, Wei Gao, Qiang Wang, Luis Augusto Rohde, Jie Zhang, Yan Li

**Affiliations:** 1 Department of Anesthesiology & Center for Brain Science, The First Affiliated Hospital of Xi’an Jiaotong University, Xi’an, Shaanxi China; 2 Children’s Health Care Center, Xi’an Children’s Hospital, Xi’an, Shaanxi China; 3 Department of Rehabilitation Medicine, The Seventh Affiliated Hospital Sun Yat-sen University, Shenzhen, Guangdong China; 4 Department of Acupuncture, Affiliated Hospital of Shaanxi University of Chinese Medicine, Xianyang, Shaanxi China; 5 ADHD and Developmental Psychiatry Programs, Hospital de Clínicas de Porto Alegre, Federal University of Rio Grande do Sul, Porto Alegre, Brazil

**Keywords:** ADHD, Human behaviour

## Abstract

Little is known about the effects of transcutaneous electrical acupoint stimulation (TEAS) for children with attention-deficit/hyperactivity disorder (ADHD). Here, we carried out a 4 week randomized clinical trial in which patients aged 6–12 years old with an ADHD diagnosis received TEAS or sham TEAS. The primary outcome measure was the investigator-rated Clinical Global Impression-Improvement (CGI-I) score at week 4. Secondary outcomes included changes from baseline to week 4 in the investigator-rated Clinical Global Impression-Severity of Illness (CGI-S) score, the Conners’ Parent/Teacher Rating Scales-Revised: Short Form (CPRS-R: S/CTRS-R: S) score, go/no-go task performance, and functional near-infrared spectroscopy (fNIRS)-based oxygenated hemoglobin level within the prefrontal cortex. At week 4, the CGI-I score indicated improvement in 33.3% of the TEAS group compared with 7.7% of the sham group (*P* = 0.005). The TEAS group had a greater decrease in the mean CGI-S score (−0.87) than the sham TEAS group (−0.28) (*P* = 0.003). A greater enhancement in the mean cerebral oxygenated hemoglobin within the prefrontal cortex was found in the TEAS group (0.099 mM mm) compared with the sham TEAS group (0.005 mM mm) (*P* < 0.001). CPRS-R: S score, CTRS-R: S score, and go/no-go performance exhibited no significant improvement after TEAS treatment. The manipulation-associated adverse events were uncommon in both groups, and events were very mild. Our results show that noninvasive TEAS significantly improved general symptoms and increased prefrontal cortex blood flow within 4 weeks for children with ADHD. Further clinical trials are required to understand the long-term efficacy in a larger clinical sample. This trial was registered on ClinicalTrials.gov (NCT 03917953).

## Introduction

Attention-deficit/hyperactivity disorder (ADHD) has a worldwide prevalence of ~5% [1, 2]. ADHD is involved in highly heterogeneous impairments in cognitive and social functions and may lead to lifelong adverse outcomes such as serious mental illness and academic failure [1, 3]. Psychostimulant and nonpsychostimulant medications are effective for reducing the main symptoms of ADHD. However, not all children respond to pharmacological treatment, and some medications have significant adverse effects [4, 5]. Hence, other alternative approaches are urgently needed.

Acupuncture or electroacupuncture is being increasingly used to manage ADHD in some countries [6–8], especially for medication-refractory patients or patients presenting intolerable adverse events with medications [9]. Transcutaneous electrical acupoint stimulation (TEAS), a noninvasive treatment, was shown to produce stimulation on acupoints similar to that of electric acupuncture [10, 11]. Consequently, TEAS is an easily accepted alternative option that has been used for children with psychiatric disorders such as autism [12, 13]. However, little is known about its efficacy on patients with ADHD.

Several studies involving neuroimaging techniques, including positron emission tomography (PET) and functional magnetic resonance imaging (fMRI), have been used to investigate the cerebral mechanism of acupuncture [14, 15]. Neuroimaging studies using PET or fMRI coupled with interventions in children with ADHD are scarce due to logistic issues, including head motion, which is a frequent problem for children with ADHD. Due to its unrestrictiveness and accessibility, functional near-infrared spectroscopy (fNIRS) has been increasingly used to assess the brain response in therapeutic protocols for children with ADHD [16, 17]. Therefore, the present clinical trial aims (1) to assess the effect of TEAS compared with that of sham TEAS in improving ADHD symptoms and (2) to explore the cerebral response to both TEAS and sham TEAS using fNIRS.

## Methods

### Study design and participants

This was a randomized, 4-week trial comparing TEAS and sham TEAS treatment for ADHD. The clinical trial was approved by the Ethics Committee of the First Affiliated Hospital of Xi’an Jiaotong University. The detailed trial protocol is provided in Supplement 1. Study enrollment started on July 1, 2019, and continued to December 1, 2019, with data collection completion on January 17, 2020. The participants and their parents provided written informed consent, respectively.

Seventy-eight children with ADHD from Xi’an Children’s Hospital were recruited and randomized for the present study. All participants fulfilled the clinical diagnostic criteria of ADHD according to the Diagnostic and Statistical Manual of Mental Disorders, 5th edition (DSM-V). The formal ADHD diagnosis was performed by two experienced child psychiatrists using clinical data and rating scales from parents, teachers, and investigators, including the Conners’ Parent Rating Scales-Revised: Short Form (CPRS-R: S), Conners’ Teacher Rating Scales-Revised: Short Form (CTRS-R: S), and Clinical Global Impression-Severity of Illness (CGI-S). The IQ of each child was evaluated by the Chinese version of the Wechsler Intelligence Scale for Children-Revised [18].

The inclusion criteria were (a) clinical formal diagnosis of ADHD and (b) age between 6 and 12 years. The exclusion criteria were (a) the presence of any other mental or neurodevelopmental disorder (e.g., comorbidities such as Autism Spectrum Disorder, Tic Disorder, Anxiety Disorder, Major Depressive Disorder, Conduct Disorder, and Oppositional Defiant Disorder) or epilepsy according to the DSM-V by clinical assessments, (b) IQ score below 75, (c) any previous acupoint-associated treatment experiences, (d) use of any ADHD medication within 1 month prior to TEAS treatment, and (e) left-handedness.

### Randomization and blinding

Eligible participants were randomly assigned in an equal ratio to undergo either TEAS or sham TEAS treatment according to a computer-generated randomization sequence. Allocation of participants was performed by a clinically independent researcher who was not involved in outcome assessment. Participants in the true and sham TEAS groups were treated in separate rooms and blinded to the intervention. The investigators who manipulated the true or sham TEAS could not be blinded to the group allocations. The psychiatrists, parents, teachers, data collectors, and statisticians were blinded to treatment assignments.

### Interventions

Following randomization, participants had an appointment with the TEAS operator. The TEAS operators had a minimum of 2 years of experience in acupuncture treatment and held a membership with a national professional association in China. The Baihui (GV 20), bilateral Taixi (KI 3) and bilateral Taichong (LR 3) acupuncture points were selected according to the concept of traditional Chinese medicine that Yin-Yang disharmony is implicated in the development of ADHD. The Baihui (GV 20) acupoint is located on the midsagittal line at the intersection of a line connecting the ear apices. The Taichong (LR 3) and Taixi (KI 3) acupoints are located on the dorsum and medial side of the foot, respectively (Fig. 2B). Self-adhesive electrodes (Supplementary Fig. 1) were attached to the children’s acupuncture points and connected to the electroacupuncture apparatus instrument (Hwato, SDZ-V, Soochow Medical Instruments Co, Ltd, Soochow, China). Dense-sparse wave alternating frequencies of 2 and 10 Hz for a 2 s cycle with an intensity of 8 ~ 10 mA, which the patients could tolerate, was administered to participants in the TEAS group. The treatment was performed once a day, twice a week. Each patient underwent eight sessions, the stimulation was 20 min per session, and there was a 2- or 3-day interval between each pair of sessions in a week. The sham TEAS group was stimulated at the same acupuncture points as those used in the TEAS group, and other intervention measures were the same for the sham TEAS group as those used in the TEAS group, except that the current intensity was set to 0 mA.

Before and after 4 weeks of TEAS or sham TEAS intervention, we used a multichannel fNIRS system (ETG-4000, Hitachi Medical Corporation, Japan) to measure the concentration changes of brain oxygenated hemoglobin (HbO) at the 695 and 830 nm wavelengths of near-infrared light. HbO signaling is more sensitive to cerebral blood flow velocity than deoxygenated hemoglobin or total hemoglobin signaling. Here, we used a 52-channel patch consisting of 17 emitters and 16 detectors (3 × 11) (Fig. 3A).

In this trial, fNIRS was used to monitor the brain response when the patients performed a go/no-go task, a computerized test that measures inhibition control [16]. The task contained six block sets, and each consisted of alternating go and go/no-go blocks. Each go or go/no-go block lasted 24 s and was preceded by a short instruction for 3 s. Therefore, a block set time was 54 s, and the full session time was ~6 min. During the go block, the participants were shown two random go images (tiger or elephant) and instructed to press a button when they caught sight of either of these images. In the go/no-go block, the patients were randomly shown a go image (lion) and no-go image (giraffe) and instructed to respond to the go image (press the button) and inhibit their response to the no-go image. The short instruction was displayed in Chinese for 3 s before each block as follows: “press the button for tiger or elephant image” in the go block and “press the button for lion image and do not press the button for giraffe image” in the go/no-go block. Patients pressed the button with the right forefinger. The images were selected as in previous neuroimaging studies [16]. Each participant was required to perform the practice blocks before any formal measurements to make sure that they completely understood the instructions (Fig. 2C).

### Outcomes

The primary outcome, the Clinical Global Impression-Improvement (CGI-I) scale, was measured at week 4 after TEAS or sham TEAS manipulation. The psychiatrists, blinded to the randomized treatment, administered the CGI-I scale to the patients at week 4. The CGI-I scale was used to assess the improvement, maintenance, or worsening of patients’ symptoms compared to baseline. The CGI-I scale contains seven levels for scoring: 1 = very much improved, 2 = much improved, 3 = minimally improved, 4 = no change, 5 = minimally worse, 6 = much worse, and 7 = very much worse [19]. The clinical manifestation of the patients at week 4 was considered to be a rating of “very much improved” or “much improved” (1 or 2), which is defined as a clinically meaningful response. We obtained CGI-I ratings at week 4 after complete treatment or at the time of dropout for participants who withdrew from the trial.

Secondary outcomes included the CGI-S score, CPRS-R: S score, CTRS-R: S score, go/no-go performance, and HbO concentration at channel 37 (CH 37) within the frontal lobe cortex at week 4 and its changes from baseline to week 4. The CGI-S is a clinical psychiatrist-rated scale that includes seven levels for scoring: 1 = not at all ill, 2 = borderline mentally ill, 3 = mildly ill, 4 = moderately ill, 5 = markedly ill, 6 = severely ill, and 7 = among the most extremely ill [20]. The CPRS-R: S and CTRS-R: S are parent- and teacher-rated standard instruments for the assessment of ADHD in children and adolescents. The two scales comprise a 27-item 4-point [21] and a 28-item 4-point [20] symptom checklist, respectively. After the go/no-go task, the mean accuracy (ACC) of go/no-go trials and reaction time (RT) of go trials were separately calculated [16]. A significant increase in HbO concentration in the specific channel from baseline to week 4 was considered an enhancement of the regional cerebral blood flow [22]. CH 37, located in the prefrontal cortex, was a priori defined as a sensitive region for discriminating children with ADHD from those with typical development [23].

### Statistical analysis

There were no studies available concerning the effect of TEAS on ADHD, to provide information about an optimal sample size. In this trial, the sample size was calculated according to the results of a pilot study (*n* = 40) that found that the CGI-I scale rated a global improvement of 45% and 5% in TEAS and sham TEAS groups, respectively. A sample size of 36 participants (18 per group) was estimated according to a priori computation using the program G*Power (version 3.1.9.2, University of Dusseldorf) with a power of at least 80% to detect a 2-sided significance level of 5%. Here, 78 participants were included to account for potential missing samples.

Statistical analyses were based on both the intention-to-treat (ITT) and planned per-protocol (PP) principles and were performed with IBM SPSS Statistics 19. The baseline characteristics of the TEAS and sham TEAS groups are described by the mean (SD). The primary outcome difference between the randomized groups was analyzed by using the chi-square test to assess the CGI-I scores for dichotomy, comparing “very much improved” and “much improved” (defined as improved) with all other ratings (defined as not improved). Secondary outcome differences between the two groups were analyzed by calculating the mean (95% CI). The group-by-time interaction of the mixed model for repeated measures (MMRM) was used to analyze differences in the secondary outcomes, with groups (TEAS vs. sham TEAS) and time (baseline vs. week 4) as individual fixed effects. We added simple effects analysis to the MMRM analysis to investigate intergroup and intragroup differences before and after treatment between the TEAS and sham TEAS groups to verify the treatment effect while ensuring a consistent experimental baseline. The significance threshold for all analyses was set at 0.05. The Bonferroni correction was applied to correct for multiple comparisons.

### Deviations from the original protocol

We made some changes to the original plan described at https://www.clinicaltrials.gov/ct2/show/NCT03917953?term=NCT+03917953&draw=2&rank=1. First, changes from baseline to week 4 in the CGI-S, CPRS-R: S, and CTRS-R: S scores were added as aspects of the secondary outcomes. Second, the diversity of the gut microbiota was omitted from the secondary outcomes. Third, a 6-month follow-up was not performed owing to the COVID-19 epidemic. We have updated these changes in the ClinicalTrials system.

## Results

### Participants and baseline characteristics

**Fig. 1:**
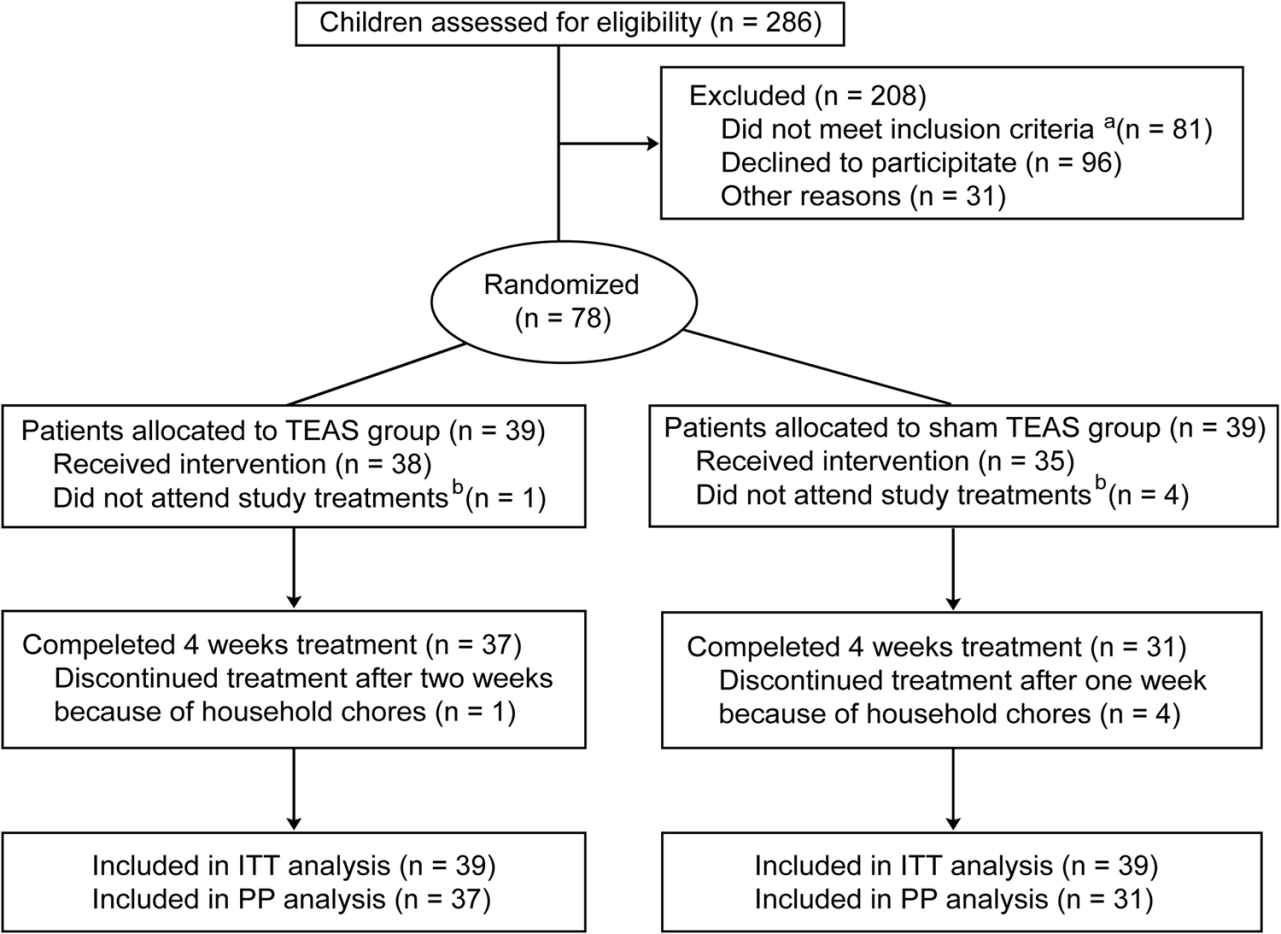
CONSORT flow diagram of participants through the trial. ^a^Reasons for children who did not meet inclusion criteria or did not attend study treatments are not available. ^b^Their residential addresses were too far from the hospital to attend this study. ADHD attention-deficit/hyperactivity disorder, TEAS transcutaneous electrical acupoint stimulation, ITT intention-to-treat, PP planned per-protocol.

**Fig. 2:**
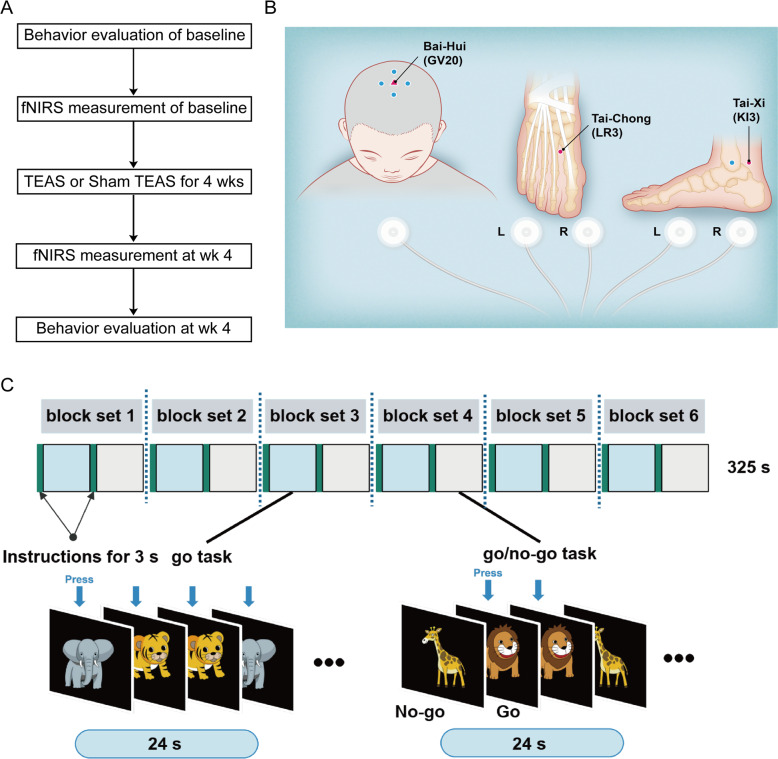
Study design. **A** Flowchart showing the study design of the TEAS intervention and response evaluation. **B** Illustration of acupoint locations for TEAS and sham TEAS. The rose-red triangle is the Baihui acupoint in the left panel, and the rose-red circles are the Taichong and Taixi acupoints in the middle and right panels, respectively. **C** Schematic diagram of the go/no-go task. HbO was measured by fNIRS in the TEAS and sham TEAS groups performing the go/no-go task.

Between July 1, 2019, and January 17, 2020, after screening 286 participants, 78 patients aged 6–12 years were randomly assigned (1:1) to receive either TEAS or sham TEAS treatment (Fig. 1). Most participants were treatment-naïve. For the 12 participants who had a medication history, four patients (three in the TEAS group and one in the sham TEAS group) had received methylphenidate. Eight patients (three in the TEAS group and five in the sham TEAS group) had received traditional Chinese medicine for ADHD. Among the randomized individuals, 68 completed the 4-week treatment, symptom evaluation, and fNIRS analyses at baseline and week 4 (Fig. 2A). Of the other ten participants, five patients (one in the TEAS group and four in the sham TEAS group) did not receive any treatment and mean imputation was used for these missing data. Five patients (1 in the TEAS group and 4 in the sham TEAS group) withdrew from the trial during week 1 or 2, and the endpoint scores of the scales and fNIRS measurement were collected at the time of dropout. We noticed a higher dropout rate in the sham TEAS group than in the TEAS group (8 vs. 2 patients). Therefore, both an ITT and a PP analysis were performed. The baseline demographic and clinical characteristics were similar between the true and sham TEAS groups (Table 1 and Supplementary Table 1).

**Table 1:** Baseline characteristics of patients in the True TEAS and Sham TEAS groups in the ITT analysis.

Characteristics	TEAS (*n* = 39)	Sham TEAS (*n* = 39)
Male, No. (%)	33 (84.6)	31 (79.5)
Age, mean (SD), year	8.05 (1.187)	8.56 (1.468)
IQ, mean (SD), score	93.90 (12.333)	96.72 (11.596)
BMI, mean (SD)	17.36 (2.743)	17.39 (3.419)
Subtype, No. (%)		
ADHD-I	17 (43.6)	19 (48.7)
ADHD-HI	0 (0)	0 (0)
ADHD-C	22 (56.4)	20 (51.3)
Ethnicity, No. (%)		
Han	39 (100)	38 (97.4)
Others	0 (0)	1 (2.6)

*TEAS* transcutaneous electrical acupoint stimulation, *ITT* intention-to-treat, *IQ* intelligence quotient, *BMI* body mass index.

### Primary outcome

ITT analysis of individual CGI-I scores indicated improved overall functioning in 13 of the 39 patients (33.3%) in the TEAS group compared with three of the 39 patients (7.7%) in the sham TEAS group, and there was a significant difference between the two groups (*x*^2^ = 7.863, df = 1, *P* = 0.005) (Table 2). The primary outcome also showed a marked difference between the TEAS and the sham TEAS in PP analysis (*x*^2^ = 6.076, df = 1, *P* = 0.014) (Supplementary Table 2).

**Table 2:** Primary and secondary outcomes from the ITT Analysis.

	TEAS (*n* = 39)	Sham TEAS (*n* = 39)	*P-*value				
Primary outcome							
CGI-I							
Score at wk 4^a^							
Improved, No. (%)	13 (33.3)	3 (7.7)	0.005				
Not improved, No. (%)	26 (66.7)	36 (92.3)					
Secondary outcomes			Interaction term	TEAS and Sham TEAS		Baseline and Week 4	
				**Baseline**	**Week 4**	**TEAS**	**Sham TEAS**
CGI-S, mean (95% CI)							
Score at baseline	4.36 (4.12–4.60)	4.66 (4.41–4.90)	0.001	0.111	<0.001	<0.001	0.025
Score at wk 4	3.49 (3.21–3.76)	4.38 (4.08–4.67)					
Change at wk 4^b^	−0.87 (−1.12−0.63)	−0.28 (−0.53−0.04)					
CPRS-R: S, mean (95% CI)							
Score at baseline	45.82 (41.96–49.68)	47.13 (44.29–49.96)	0.384	0.639	0.220	<0.001	0.003
Score at wk 4	38.36 (33.49–43.22)	41.78 (37.71–45.85)					
Change at wk 4^c^	−7.46 (−10.87−4.06)	−5.34 (−8.75−1.94)					
CTRS-R: S, mean (95% CI)							
Score at baseline	31.28 (26.37–36.19)	35.28 (31.17–39.39)	0.956	0.239	0.222	0.291	0.328
Score at wk 4	29.26 (23.86–34.65)	33.41 (28.56–38.25)					
Change at wk 4^d^	−2.03 (−5.82–1.77)	−1.88 (−5.67–1.92)					
ACC (%), mean (95% CI)							
At baseline	83.96 (79.71–88.21)	84.97 (82.36–87.58)	0.049	0.639	0.084	<0.001	0.196
At wk 4	90.86 (88.17–93.56)	87.14 (84.84–89.45)					
Change at wk 4^e^	6.90 (3.58–10.22)	2.18 (−1.14–5.49)					
RT (ms), mean (95% CI)							
At baseline	304.2 (274.1–334.4)	318.1 (286.9–349.2)	0.193	0.620	0.252	0.016	0.551
At wk 4	365.0 (324.8–405.1)	332.9 (279.2–386.5)					
Change at wk 4^f^	60.74 (11.5–109.9)	14.8 (−34.4–64.0)					
Oxy-HB CH 37 (mM mm), mean (95% CI)							
At baseline	0.021 (0.007–0.049)	0.024 (0.009–0.040)	0.001	0.881	<0.001	<0.001	0.812
At wk 4	0.120 (0.078–0.162)	0.029 (0.005–0.062)					
Change at wk 4^g^	0.099 (0.061–0.138)	0.005 (−0.034–0.043)					

*ITT* intention-to-treat, *TEAS* transcutaneous electrical acupoint stimulation, *CGI*-*I* Clinical Global Impression Scale-Improvement of Illness, *CGI*-*S* Clinical Global Impression-Severity of Illness, *CPRS*-*R*: *S* Conners’ Parent Rating Scales-Revised: Short Form, *CTRS*-*R*: *S*, Conners’ Teacher Rating Scales-Revised: Short Form, *ACC* accuracy, *RT* reaction time, *Oxy*-*Hb* oxygenated hemoglobin, *SD* standard deviation.

^a^Chi-square test to assess the CGI-I scores at week 4.

^b–g^Indicates the difference in mean change from baseline to endpoint between the TEAS and sham TEAS groups by MMRM and the Wilcoxon rank-sum test. The main effect for time was significant for the ^b^CGI-S score (*Z* = −2.955, *P* = 0.003) and ^g^Oxy-HB CH 37 (*Z* = −4.464, *P* < 0.001) but not for the ^c^CPRS-R: S score (*Z* = −0.520, *P* = 0.603), ^d^CTRS-R: S score (*Z* = −0.313, *P* = 0.754), ^e^ACC (*Z* = −1.865, *P* = 0.062), and ^f^RT (*Z* = −1.520, *P* = 0.129).

### Secondary outcomes

MMRM analyses in the ITT population (Table 2 and Supplementary Table 3) showed significant differences in the group-by-time interaction effect between the TEAS and sham TEAS groups in the mean CGI-S score (*P* = 0.001), ACC for go/no-go trials (*P* = 0.049), and HbO concentration for CH 37 of fNIRS (*P* = 0.001) but not in the mean CPRS-R: S score (*P* = 0.384), CTRS-R: S score (*P* = 0.956), or RT for go trials (*P* = 0.193).

The mean CGI-S score was 4.36 (95% CI, 4.12–4.60) at baseline and 3.49 (95% CI, 3.21–3.76) at week 4 in the TEAS group and 4.66 (95% CI, 4.41–4.90) at baseline and 4.38 (95% CI, 4.08–4.67) at week 4 in the sham TEAS group. There was no significant between-group difference in the mean CGI-S score at baseline (*P* = 0.111). The reduction in the CGI-S score from baseline to week 4 was greater in the TEAS group (mean, −0.87) than in the sham TEAS group (mean, −0.28) (*P* = 0.003).

At baseline, the mean CPRS-R: S score was 45.82 (95% CI, 41.96–49.68) in the TEAS group and 47.13 (95% CI, 44.29–49.96) in the sham TEAS group. At week 4, the mean CPRS-R: S scores were 38.36 (95% CI, 33.49–43.22) and 41.78 (95% CI, 37.71–45.85) in the TEAS and sham TEAS groups, respectively. Although the mean CPRS-R: S scores in both the TEAS (*P* < 0.001) and sham TEAS (*P* = 0.003) groups at week 4 were greater than those at baseline, no significant difference in the mean change over 4 weeks was found between the two groups (*P* = 0.603). The mean CTRS-R: S score was 31.28 (95% CI, 26.37–36.19) at baseline and 29.26 (95% CI, 23.86–34.65) at week 4 in the TEAS group and 35.28 (95% CI, 31.17–39.39) at baseline and 33.41 (95% CI, 28.56–38.25) at week 4 in the sham TEAS group. No difference was found between the two groups in the mean CTRS-R:S scores after true or sham TEAS treatment.

In addition, the mean ACC for go/no-go trials was 83.96% (95% CI, 79.71–88.21) at baseline and 90.86% (95% CI, 88.17–93.56) at week 4 in the TEAS group and 84.97% (95% CI, 82.36–87.58) at baseline and 87.14% (95% CI, 84.84–89.45) at week 4 in the sham TEAS group. The mean RT for go/no-go trials was 304.2 ms (95% CI, 274.1–334.4) at baseline and 365.0 ms (95% CI, 324.8–405.1) at week 4 in the TEAS group and 318.1 ms (95% CI, 286.9–349.2) at baseline and 332.9 ms (95% CI, 279.2–386.5) at week 4 in the sham TEAS group. Despite increased mean ACC and RT in the TEAS group at week 4 compared with those at baseline (*P* < 0.001 and *P* = 0.016), there was no significant difference between the TEAS and sham TEAS groups at week 4 (*P* = 0.084 and *P* = 0.252).

**Fig. 3:**
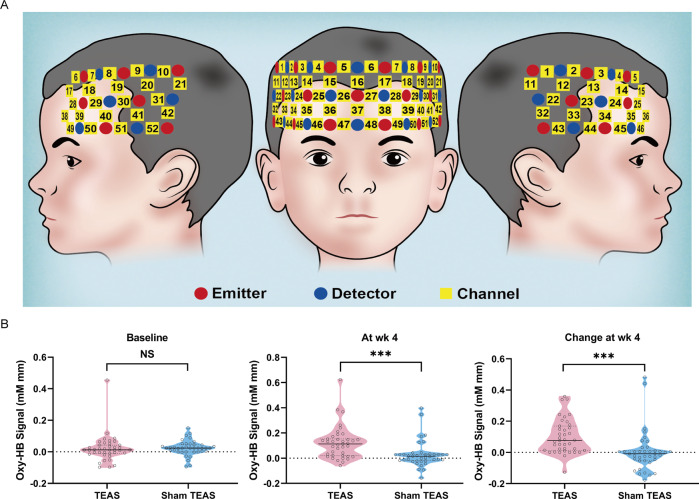
Spatial profiles of fNIRS channels and HbO signals for CH 37. **A** Map of 52-channel fNIRS used in the present trial. Each yellow square on the standard brain model represents an fNIRS channel, each red dot represents an emitter, and each blue dot represents a detector. Left-, front- and right-side views of the probe arrangements in a 52-channel fNIRS. **B** Violin plots of HbO signals for CH 37 showed no significant differences at baseline between the TEAS (*n* = 39) and sham TEAS (*n* = 39) groups (left panel) but increased brain responses were observed after the 4 weeks of treatment in the TEAS group compared to the sham TEAS group (middle and right panels) (MMRM and Wilcoxon rank-sum test). Hollow circles denote individual data points, solid black lines denote medians, and dashed gray lines denote quartiles. NS not significant. ***p < 0.001.

fNIRS was used to assess the cerebral blood flow response to TEAS by monitoring HbO concentrations. CH 37, located in the frontal lobe cortex (Fig. 3A), was previously identified as an effective fNIRS channel involved in the go/no-go task and showed a significantly higher HbO concentration in children with typical development than in children with ADHD [23]. The mean baseline HbO concentrations for CH 37 were 0.021 mM mm (95% CI, 0.007–0.049) and 0.024 mM mm (95% CI, 0.009–0.040) in the TEAS group and the sham TEAS group, respectively (*P* = 0.881). We found a higher mean HbO signal in the ADHD subjects who received TEAS (0.120 mM mm; 95% CI, 0.078–0.162) than in the ADHD individuals in the sham TEAS group (0.029 mM mm; 95% CI, 0.005–0.062) at week 4 (*P* < 0.001; Table 2 and Fig. 3B). However, no changes were found in the sham TEAS group after 4 weeks of treatment (*P* = 0.812). We simultaneously analyzed the other 51 channels and did not find significant differences in the concentration of HbO in these channels between the TEAS and sham TEAS groups after the Bonferroni correction (Supplement 3 and Supplement 4).

MMRM analyses in the PP population (Supplementary Table 2 and Supplementary Table 4) showed differences similar to those in the ITT population.

### Adverse events

The guardians of the patients were interviewed about the potential adverse effects of every treatment. Three participants (2 in the TEAS group and 1 in the sham TEAS group) reported adverse events during the treatment period. One patient from the TEAS group complained of skin itching at the right ankle where electrodes were attached. The other patient in the TEAS group had a sleep disorder that mainly manifested as difficulty initiating sleep. The patient in the sham TEAS group described a mood disturbance. None of the three individuals required additional medical interventions for these adverse events and did not withdraw from the entire trial.

## Discussion

To our knowledge, this is the first randomized clinical trial of TEAS in patients with ADHD. The primary outcomes from both ITT and PP analyses showed a marked difference between the TEAS and sham TEAS groups, and the secondary outcome from the ITT population showed significant differences in the mean CGI-S score, ACC for go/no-go trials, and HbO concentration for CH 37 of fNIRS but not in the mean CPRS-R: S score, CTRS-R: S score, or RT for go trials between the TEAS and sham TEAS groups. Among children with ADHD, treatment with TEAS compared with sham TEAS resulted in a significant investigator-rated clinical improvement in the ADHD-associated symptoms at week 4. In addition, patients who under TEAS showed a greater brain response monitored by fNIRS in the prefrontal cortex than individuals under sham TEAS. Very mild adverse effects were found after the intervention.

Here, patients received TEAS or sham TEAS at the Baihui, Taichong, and Taixi acupoints. Stimulation at these points is expected to harmonize the mind and body, and these points have been frequently stimulated alone or in combination with other acupoints in ADHD treatment [6, 7, 24, 25]. Acupuncture at the Baihui acupoint can increase hippocampal and cortical dopamine levels, which might partly mimic the mechanism of medications [26]. Taichong has been practiced to calm excessive Yang [27], and Taixi has been practiced to rectify insufficient Yin [7, 27]. Previous acupuncture or electroacupuncture studies showed improvement in ADHD behaviors [7, 27] or increases in learning/memory abilities [25]. However, most of these acupoint-associated trials were not strict randomized controlled trials.

We can obtain specific improvement information from the CGI-I scale as it includes seven options for scoring, and a level of “much” or “very much improved” (score of 1 or 2) is defined as a clinically meaningful response. Therefore, this measure requires the psychiatrist to assess whether children’s behaviors have improved or worsened at the end of TEAS or sham TEAS treatment according to their symptoms at the beginning of treatment. We found that 4 weeks of TEAS significantly relieved the general symptoms of patients compared with sham TEAS. Here, the decrease in CGI-S scores at week 4 from baseline was remarkable in the TEAS group compared with that in the sham TEAS group.

However, the changes over 4 weeks in scores on the CPRS-R: S and CTRS-R: S, two rating scales relying on parent and teacher reports, did not show prominent differences between the true and sham TEAS groups, although the mean CPRS-R: S scores in both the TEAS and sham TEAS groups at week 4 were greater than those at baseline. Therefore, there may be moderate placebo effects produced by sham TEAS. We noticed a discrepancy between measurements from clinical psychiatrists and parents/teachers. On one hand, this might suggest that investigators and parents/teachers might have different perspectives on children’s ADHD symptoms. CGI-I and CGI-S scores provide a general impression according to patients’ integrated behaviors, while the CPRS-R: S and CTRS-R: S assessments require more focused evaluation of specific ADHD symptoms. On the other hand, and more importantly, we acknowledge the large numbers of parents and teachers (a fifth to a quarter) who finished the online measurements in a very short time at week 4 after the trial. Thus, it is difficult to guarantee the accuracy and quality of those CPRS-R: S and CTRS-R: S evaluations. We noticed that the MMRM in the ITT population (Table 2) showed a significant difference in the group-by-time interaction effect between the TEAS and sham TEAS groups in the mean ACC for go/no-go trials (*p* = 0.049), which is very close to 0.05. However, the PP analysis (Supplementary Table 2) showed no significant difference in the group-by-time interaction effect between the TEAS and sham TEAS groups in the mean ACC (*p* = 0.067).

Acupuncture is known to increase local cerebral blood flow [28, 29], although the mechanisms of its efficacy for ADHD are still largely unknown [30]. fNIRS has been more commonly used to assess brain functioning in infants and children because of its accessibility and its ability to provide valuable results in spite of body movement [31, 32]. Although its detection space is limited to superficial cortical regions of the brain, fNIRS is a viable brain imaging tool for children with ADHD after weighing the pros and cons of the technology [33, 34]. ADHD is associated with dysfunction of the frontostriatal network [35, 36]. In this trial, we used a 3 × 11 probe (52 channels) system to monitor cerebral responses before and after TEAS for patients with ADHD [23, 37]. The neuroimages of children with ADHD, adolescents, and adults showed that right middle frontal activation is distinctly associated with response inhibition dysfunction [38]. CH 37, located in the prefrontal cortex, was a priori defined as a sensitive region for discriminating children with ADHD from children with typical development [23]. Interestingly, 4 weeks of TEAS significantly increased the concentration of HbO in CH 37 in patients during the go/no-go task but not in individuals who underwent sham TEAS. Acupuncture at the Baihui acupoint can increase the dopamine levels in the cerebral cortex, which might partly mimic the mechanism of medications [26]. Therefore, we hypothesized that CH 37 represented not only a sensitive cortex for ADHD diagnosis but also a cortex that is responsive to TEAS in this trial. Moreover, we simultaneously analyzed the other 51 channels and did not find significant differences in the concentration of HbO in these channels between the TEAS and sham TEAS groups. The fNIRS measurement was performed 2 h after the last TEAS treatment on the same day. TEAS has a cumulative effect according to the concept of traditional Chinese medicine [13]. Therefore, the influence of acute effects after TEAS needs to be assessed by further follow-up visits. Moreover, to explore the effects of TEAS on the routine development of children, some children with typical development should be recruited to undergo TEAS treatment and detection of the cerebral blood flow using fNIRS and fMRI before and after TEAS in a future study.

Owing to its noninvasive feature, TEAS is an easily acceptable treatment for pediatric patients with ADHD. Parents might be easily taught to administer TEAS to children with ADHD at home. The patients most likely to benefit from TEAS include those who are intolerant or do not respond to psychostimulants. We noticed that a number of qualified patients refused to participate in our trial because their previous medication treatments worked very well. In addition, although inferior to TEAS, sham TEAS still exhibited a slight improvement, and this effect was most likely a result of the placebo effect originating from routine manipulation. Second, the electrodes without electricity may not be completely inert. In the trial, we used an electrode with a small bulge in the middle to match the acupoints (Supplementary Fig. 1).

Of note, this study has several limitations. First, the sample size was moderate since participants were enrolled from a single medical center. Second, only the clinical investigator-rated CGI-I score was used as the primary outcome, and integrated measures for ADHD from psychiatrists and parents/teachers were not included as primary outcomes. Third, it is not known whether all three acupoints contribute to improved behaviors. Finally, this was only a cross-sectional study at baseline and week 4, without longitudinal assessments.

Overall, TEAS can be safely practiced on children with ADHD. Compared with sham TEAS, TEAS resulted in a larger general symptomatic improvement in patients and greater prefrontal responses within 4 weeks of administration. Further clinical trials are required to understand the long-term benefits of TEAS for children with ADHD, especially for those who are intolerant or have no response to routine psychostimulant therapy.

## Supplementary information

Supplement 1

Supplement 2

Supplement 3

Supplement 4
