# Supplementary material for: Transcutaneous electrical acupoint stimulation for children with attention-deficit/hyperactivity disorder: a randomized clinical trial
Source: Transl Psychiatry. 2022 Apr 21;12:165. doi: 10.1038/s41398-022-01914-0 (PMC9022403; doi:10.1038/s41398-022-01914-0)
Supplement: Supplementary file 1 — Supplement 1 [file 41398_2022_1914_MOESM1_ESM.pdf]

Effect of Transcutaneous Electrical Acupoint Stimulation on Children with Attention-Deficit/Hyperactivity Disorder

RESEARCH PROTOCOL

March 2019

PROTOCOL TITLE Effect of Transcutaneous Electrical Acupoint Stimulation on Children with Attention-Deficit/Hyperactivity Disorder

**Protocol ID**	**XJTU1AF-CRF-2018-003**
**Short title**	**A Randomized Clinical Trial**
**Version**	**2**
**Date**	**March 2019**
**Project leader**	Yan Li, PhD<br>Department of Anesthesiology & Center for Brain Science,<br>The First Affiliated Hospital of Xi'an Jiaotong University,<br>Xi'an, Shaanxi 710061, China<br>liyanxjtu@xjtu.edu.cn<br><br>Jie Zhang, MD<br>Children's Health Care Center, Xi'an Children's Hospital,<br>Xi'an, Shaanxi 710003, China<br>zhangjiexach@xjtu.edu.cn
**Investigators group**	Lixia Zhuo, MD<br>Department of Anesthesiology & Center for Brain Science,<br>The First Affiliated Hospital of Xi'an Jiaotong University,<br>Xi'an, Shaanxi 710061, China<br>18292866500@163.com<br>Xiaoyan Zhao, MD<br>Children's Health Care Center, Xi'an Children's Hospital,<br>Xi'an, Shaanxi 710003, China<br>1316334110@qq.com<br>Yifang Zhai, PhD<br>Department of Anesthesiology & Center for Brain Science,<br>The First Affiliated Hospital of Xi'an Jiaotong University,<br>Xi'an, Shaanxi 710061, China<br>437171110@qq.com<br>Boqiang Zhao, MD<br>Department of Anesthesiology & Center for Brain Science,<br>The First Affiliated Hospital of Xi'an Jiaotong University,<br>Xi'an, Shaanxi 710061, China<br>zhaoboqiang@stu.xjtu.edu.cn<br>Lin Tian, MD<br>Children's Health Care Center, Xi'an Children's Hospital,<br>Xi'an, Shaanxi 710003, China<br>393661167@qq.com<br>Yannan Zhang, MD<br>Children's Health Care Center, Xi'an Children's Hospital,<br>Xi'an, Shaanxi 710003, China<br>767833509@qq.com

Xiaodan Wang, MD
Department of Anesthesiology & Center for Brain Science, The First Affiliated Hospital of Xi’an Jiaotong University, Xi’an, Shaanxi 710061, China
m19834513386@163.com

Tingyu Zhang, MD
Department of Rehabilitation Medicine, The Seventh Affiliated Hospital Sun Yat-sen University, Shenzhen, Guangdong 518107, China
katezhang@outlook.com

Xinyi Gan, MD
Department of Anesthesiology & Center for Brain Science, The First Affiliated Hospital of Xi’an Jiaotong University, Xi’an, Shaanxi 710061, China
1561041016@qq.com

Cheng Yang, MD
Department of Anesthesiology & Center for Brain Science, The First Affiliated Hospital of Xi’an Jiaotong University, Xi’an, Shaanxi 710061, China
13201586615@163.com

Weigang Wang, MD
Department of acupuncture, Affiliated Hospital of Shaanxi University of Chinese Medicine. Xianyang, Shaanxi 712000, China
332062219@qq.com

Wei Gao, MD
Department of Anesthesiology & Center for Brain Science, The First Affiliated Hospital of Xi’an Jiaotong University, Xi’an, Shaanxi 710061, China
gaowei2906@xjtufh.edu.cn

Qiang Wang, MD
Department of Anesthesiology & Center for Brain Science, The First Affiliated Hospital of Xi’an Jiaotong University, Xi’an, Shaanxi 710061, China
dr.wangqiang@139.com

Luis Augusto Rohde, MD
Department of Child and Adolescent Psychiatry, Hospital de Clínicas de Porto Alegre and Federal University of Rio Grande do Sul, Porto Alegre, Rio Grande do Sul 95035-003, Brazil
lrohde@terra.com.br

PROTOCOL SIGNATURE SHEET

Name	Signature	Date
Head of Department<br><br>Prof. Yan Li, Center for Brain Science<br>Dr. Jie Zhang, Child Healthcare	Yan Li<br>Jie Zhang	03/01/2019<br>03/01/2019
Project leader<br><br>Prof. Yan Li, Center for Brain Science<br>Dr. Jie Zhang, Child Healthcare	Yan Li<br>Jie Zhang	03/01/2019<br>03/01/2019

TABLE OF CONTENTS

PROTOCOL SIGNATURE SHEET 4
STUDY SYNOPSIS 6
1. Background of Project and Scientific Justification 7
2. Methods 7
Purpose 7
Study Design 7
  Trial Centers 7
  Subjects - Numbers and Enrollment 7
  Subjects - Definition of ADHD 7
  Subjects - Inclusion criteria 8
  Subjects - Exclusion criteria 8
Study Treatment 8
Study Evaluation 8
  CPRS-R: S 9
  CTRS-R: S 10
  The Clinical Global Impression-Improvement scale (CGI-I) 11
  The Clinical Global Impression-Severity scale (CGI-S) 12
3. Study Procedure 13
Procedures 13
  Study Procedure Checklist 14
Outcome Measures 15
  Primary outcome (measured at baseline and 4 weeks) 15
  Secondary outcomes (measured at baseline and 4 weeks) 15
4. Statistical Analysis 15
5. Consent Documentation 16

STUDY SYNOPSIS

Protocol Number (Clinical Trial Registration Identifier):
ClinicalTrials.gov ID: NCT03917953 at
https://clinicaltrials.gov/ct2/results?cond=&term=03917953&cntry=&state=&city=&dist=

Protocol Version: Version 2

Protocol Title: Effect of Transcutaneous Electrical Acupoint Stimulation on Children with Attention-Deficit/Hyperactivity Disorder

Chief Investigators: Professor Yan Li, Dr. Jie Zhang, Dr. Lixia Zhuo, Dr. Xiaoyan Zhao, Dr. Yifang Zhai

Ethics and dissemination: The study was approved by local ethic committee of human research at the First Affiliated Hospital of Xi'an Jiaotong University in China. The experimental procedure was in accordance with the ethical principles of the 1964 Declaration of Helsinki (World Medical Organization, 1996).

Name of funding source or sponsor: This trial was supported by the National Natural Science Foundation of China (No. 82071538), the Natural Science Fund for Distinguished Young Scholars of Shaanxi Province (No. 2019JC-24), the Fundamental Research Funds for the Central Universities of China (No. xtr022019005), and the Clinical Research Award of the First Affiliated Hospital of Xi'an Jiaotong University (No. XJTU1AF-CRF-2018-003).

Study Aim: To determine the efficacy and safety of TEAS in the treatment of children with ADHD and explore the cerebral response to this noninvasive treatment compared to sham TEAS.

Study Population: Children aged between 6 and 12 years with an attention deficit/hyperactivity disorder.

Study Sites: Xi'an Children's Hospital (Xi'an) and the First Affiliated Hospital of Xi'an Jiaotong University (Xi'an).

Study Design: Randomized, 4-week (8 sessions) trial comparing TEAS and sham TEAS treatment for ADHD.

Study Treatment: Patients were randomly assigned to undergo 8 sessions of TEAS at 3 traditional acupoints or sham TEAS at the same acupoints without electrical stimulation over 4 weeks.

Number of Subjects: 78 subjects randomized into two groups (TEAS and sham TEAS).

1. Background of Project and Scientific Justification

Attention deficit/hyperactivity disorder (ADHD) is a common neurodevelopmental disorder, primarily characterized with inattention and hyperactivity. Psychostimulant and non-psychostimulant medications are effective for reducing the core symptoms of ADHD. However, not all children respond to pharmacological treatment, and some medications display significant adverse effects. Hence, other alternative approaches are strongly welcomed. Transcutaneous electrical acupoint stimulation (TEAS), a noninvasive treatment, was proved to take a similar stimulation on acupoints as that of electric acupuncture. However, little is known its efficacy on patients with ADHD.

2. Methods

Purpose

Primary Purpose: To determine the efficacy and safety of TEAS in the treatment of children with ADHD and explore the cerebral response to this noninvasive treatment compared to sham TEAS.

Hypothesis: TEAS is effective compared to sham TEAS in the treatment for children with ADHD.

Study Design

This study is a randomized, 4-week (8 sessions) trial comparing TEAS and sham TEAS treatment for ADHD.

Trial Centers

The trail will take place at Xi'an Children's Hospital (Xi’an), and the data will be analyzed at the First Affiliated Hospital of Xi’an Jiaotong University (Xi’an).

Subjects - Numbers and Enrollment

Enrollment of 78 children with ADHD will take place at a single site: Xi’an Children's Hospital. Participants will be recruited through the child psychiatrists in Xi’an Children's Hospital. Professionals will receive a letter explaining the study, along with a study synopsis and copies of the study brochure. Professionals that identify appropriate participants will be asked to assist recruitment by informing families about the study and providing a general introductory letter to the families about the study. If they agree with the study treatment, they will sign an "informed consent form".

Subjects - Definition of ADHD

All participants fulfilled the clinical diagnostic criteria of ADHD according to the Diagnostic and Statistical Manual of Mental Disorders (DSM-V) Fifth Edition, was validated by two experienced child psychiatrists using rating scales from parent, teacher, and investigator, including Conners’ Parent Rating Scales-Revised: Short Form (CPRS-R: S), Conners’ Teacher Rating Scales-Revised: Short Form (CTRS-R: S), and Clinical Global Impressions Scale-Severity of Illness (CGI-S).

Subjects - Inclusion criteria

- Males and females aged 6 to 12 years.
- A diagnosis of ADHD based on the Diagnostic and Statistical Manual (DSM-V) criteria for Attention deficit/hyperactivity disorder (as above).
- Will be able to comply with the assessments and procedures required for the trial.

Subjects - Exclusion criteria

- A known DSM-V diagnosis of Rett's Disorder, Autism Spectrum Disorder, Tic Disorder or ADHD combined with other mental and neurodevelopmental disorder.
- The IQ score of the participants is below 75.
- Patients currently have received prior TEAS or other acupoints-associated treatment experiences.
- The left handedness.

Study Treatment

Patients were randomly assigned to undergo 8 sessions of TEAS at 3 traditional acupoints or sham TEAS at the same acupoints without electrical stimulation over 4 weeks.

Study Evaluation

CPRS-R: S and CTRS-R: S were measured by the parents and teachers online, respectively. CGI-I and CGI-S were evaluated by the psychiatrists at the hospital clinic.

CPRS-R: S

Name： ____________ **Sex：** ________ **Birthday：** ____Y____M____D **Grade：** _______

Respondent：Mother ☐ **； Farther** ☐ **； Grandparents** ☐ **； Other guardians** ☐

The following is a description of your kids' performance since the last interview. Please read it carefully and choose the answer appropriate for your kids' situation.

	Never	Sometimes	Often	Always
1. Inattentive	0 ☐	1 ☐	2 ☐	3 ☐
2. Angry	0 ☐	1 ☐	2 ☐	3 ☐
3. Difficulty with completing homework	0 ☐	1 ☐	2 ☐	3 ☐
4. Always on the go	0 ☐	1 ☐	2 ☐	3 ☐
5. Short attention span	0 ☐	1 ☐	2 ☐	3 ☐
6. Argues with adults	0 ☐	1 ☐	2 ☐	3 ☐
7. Fidgets	0 ☐	1 ☐	2 ☐	3 ☐
8. Fails to complete assignments	0 ☐	1 ☐	2 ☐	3 ☐
9. Hard to control in malls	0 ☐	1 ☐	2 ☐	3 ☐
10. Disorganised	0 ☐	1 ☐	2 ☐	3 ☐
11. Loses temper	0 ☐	1 ☐	2 ☐	3 ☐
12. Needs close supervision	0 ☐	1 ☐	2 ☐	3 ☐
13. Only attends if interested	0 ☐	1 ☐	2 ☐	3 ☐
14. Runs about	0 ☐	1 ☐	2 ☐	3 ☐
15. Distractible	0 ☐	1 ☐	2 ☐	3 ☐
16. Irritable	0 ☐	1 ☐	2 ☐	3 ☐
17. Difficulty with sustained task	0 ☐	1 ☐	2 ☐	3 ☐
18. Restless	0 ☐	1 ☐	2 ☐	3 ☐
19. Gets distracted	0 ☐	1 ☐	2 ☐	3 ☐
20. Refuses to comply	0 ☐	1 ☐	2 ☐	3 ☐
21. Has trouble concentrating	0 ☐	1 ☐	2 ☐	3 ☐
22. Has difficulty in turn taking	0 ☐	1 ☐	2 ☐	3 ☐
23. Leaves seat when not expected	0 ☐	1 ☐	2 ☐	3 ☐
24. Annoys people	0 ☐	1 ☐	2 ☐	3 ☐
25. Does not finish chores	0 ☐	1 ☐	2 ☐	3 ☐
26. Difficulty playing quietly	0 ☐	1 ☐	2 ☐	3 ☐
27. Easily frustrated	0 ☐	1 ☐	2 ☐	3 ☐

Total Score： ______________

Assessor's signature: _________________ **Date：** _____Y_____M_____D

CTRS-R: S

Name： ____________ **Sex：** ________ **Birthday：** ____Y____M____D **Grade：** _______

Respondent：Mother ☐ **； Farther** ☐ **； Grandparents** ☐ **； Other guardians** ☐

The following is a description of your kids' performance since the last interview. Please read it carefully and choose the answer appropriate for your kids' situation.

	Never	Sometimes	Often	Always
1. Inattentive	0 ☐	1 ☐	2 ☐	3 ☐
2. Defiant	0 ☐	1 ☐	2 ☐	3 ☐
3. Restless	0 ☐	1 ☐	2 ☐	3 ☐
4. Forgets things he has already learned	0 ☐	1 ☐	2 ☐	3 ☐
5. Disturbs other children	0 ☐	1 ☐	2 ☐	3 ☐
6. Argues with adults	0 ☐	1 ☐	2 ☐	3 ☐
7. Always on the go	0 ☐	1 ☐	2 ☐	3 ☐
8. Poor in spelling	0 ☐	1 ☐	2 ☐	3 ☐
9. Cannot remain still	0 ☐	1 ☐	2 ☐	3 ☐
10. Spiteful/vindictive	0 ☐	1 ☐	2 ☐	3 ☐
11. Leaves seat when not required	0 ☐	1 ☐	2 ☐	3 ☐
12. Fidgets	0 ☐	1 ☐	2 ☐	3 ☐
13. Not reading up to par	0 ☐	1 ☐	2 ☐	3 ☐
14. Short attention span	0 ☐	1 ☐	2 ☐	3 ☐
15. Argues with adults	0 ☐	1 ☐	2 ☐	3 ☐
16. Only attends if interested	0 ☐	1 ☐	2 ☐	3 ☐
17. Difficulty in turn taking	0 ☐	1 ☐	2 ☐	3 ☐
18. Lacks interest in schoolwork	0 ☐	1 ☐	2 ☐	3 ☐
19. Distractible	0 ☐	1 ☐	2 ☐	3 ☐
20. Temper outbursts	0 ☐	1 ☐	2 ☐	3 ☐
21. Runs about excessively	0 ☐	1 ☐	2 ☐	3 ☐
22. Poor in arithmetic	0 ☐	1 ☐	2 ☐	3 ☐
23. Interrupts other children’s activities	0 ☐	1 ☐	2 ☐	3 ☐
24. Difficulty playing quietly	0 ☐	1 ☐	2 ☐	3 ☐
25. Fails to finish chores	0 ☐	1 ☐	2 ☐	3 ☐
26. Fails to finish schoolwork	0 ☐	1 ☐	2 ☐	3 ☐
27. Excitable/impulsive	0 ☐	1 ☐	2 ☐	3 ☐
28. Restless/always on the go				

Total Score： ______________

Assessor's signature: _________________ **Date：** _____Y_____M_____D

The Clinical Global Impression-Improvement scale (CGI-I)

Name：___________ Sex：________ Birthday：____Y____M____D Grade：_______

Degree of improvement: ☐

1 = Very much improved;

2 = Much improved;

3 = Minimally improved;

4 = No change;

5 = Minimally worse;

6 = Much worse;

7 = Very much worse.

Total Score： ________________

Assessor's signature: ________ Date：___Y____M___D

The Clinical Global Impression- Severity scale (CGI-S)

**Name**：___________ **Sex**：________ **Birthday**：____Y____M____D **Grade**：_______

Disorder severity: ☐

1 = Normal, not at all ill;

2 = Borderline mentally ill;

3 = Mildly ill;

4 = Moderately ill;

5 = Markedly ill;

6 = Severely ill;

7 = The most extremely ill patients.

Total Score： _______________

Assessor's signature: ________ **Date**： _____Y_____M_____D

3. Study Procedure

Procedures

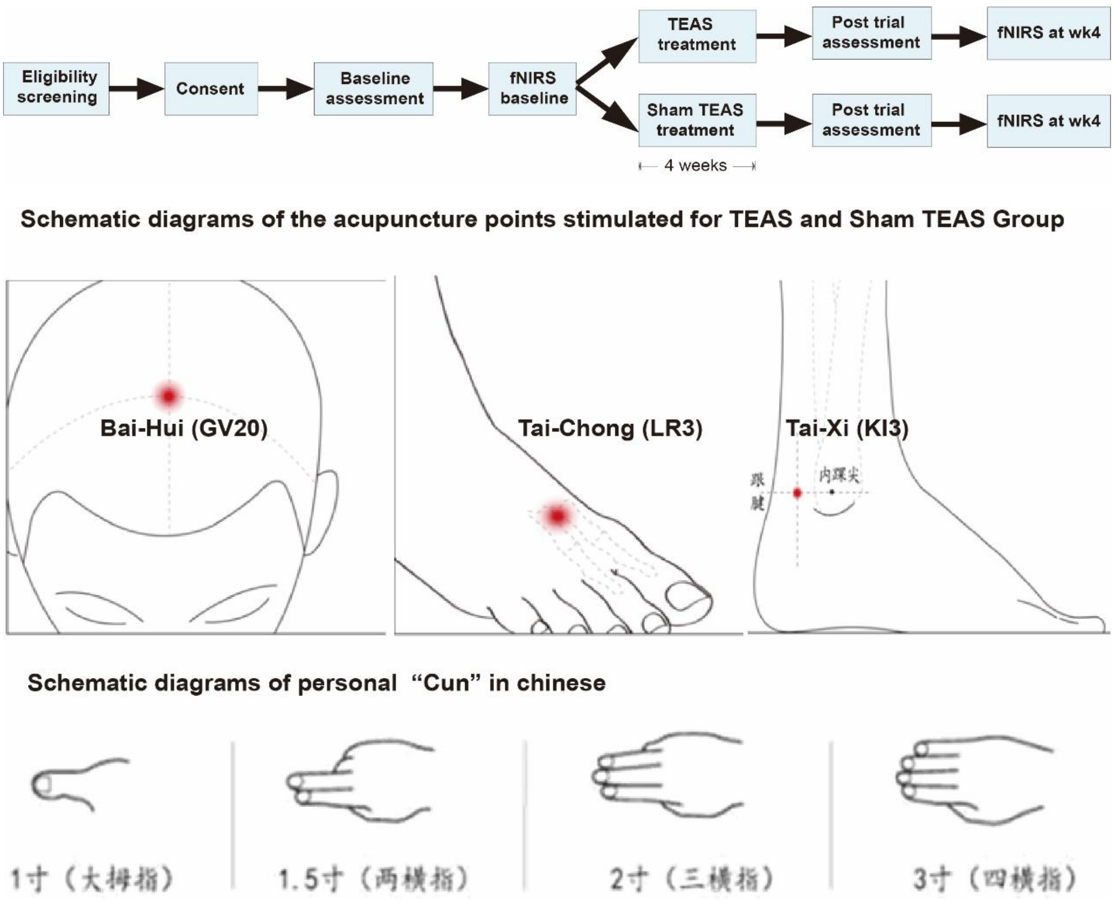

For TEAS, Bai-Hui (GV 20), bilateral Tai-Xi (KI 3) and bilateral Tai-Chong (LR 3) acupuncture points were selected according to traditional Chinese medicine theories that ADHD is caused by Yin-Yang disharmony. The self-adhesive electrodes were attached to the children’s acupuncture points and connected to the electroacupuncture apparatus instrument (Hwato, model No. SDZ-V, Suzhou Medical Instruments Co, Ltd, Suzhou, China) and selected the dense-sparse wave type with an intensity of 8 ~ 10 mA which the patients could tolerate and frequency of 2/10 Hz for four weeks for 20 min/day. The treatment was once a day, twice a week, each patient undertook 8 sessions and there was a 2 or 3-day interval between each two sessions. The sham TEAS group selected the same acupuncture points with the TEAS group and other intervention measures were the same as the TEAS group except for the current intensity was set to 0 mA.

Study Procedure Checklist

Procedure	Initial Screening	Testing [Pre]	Week1 Session1/2	Week2 Session3/4	Week3 Session5/6	Week 4 Session7/8	Testing [Post]
Inc & Exc Criteria	√						
Consent		√					
Randomization		√					
Demographic data		√					
Physical examination		√					
IQ Testing		√					
Trial Questionnaires		√					
CPRS-R: S		√					√
CTRS-R: S		√					√
CGI-I							√
CGI-S		√					√
Go/no-go task		√					√
fNIRS examination		√					√
Side Effects Checklist			√	√	√	√	√

Outcome Measures

Primary outcome (measured at baseline and 4 weeks)

The Clinical Global Impression-Improvement (CGI-I) scale was used to assess the clinical response at week 4 after TEAS or sham TEAS manipulation. The CGI-I Scale includes seven options for scoring: 1 very much improved; 2 much improved; 3 minimally improved; 4 no change; 5 minimally worse; 6 much worse; and 7 very much worse. Clinical response at week 4 was defined as a rating of “much” or “very much improved” (1 or 2), which is considered a clinically meaningful response.

Secondary outcomes (measured at baseline and 4 weeks)

The Clinical Global Impression-Severity (CGI-S) scale was used to assess the clinical response at week 4 and its changes from baseline to week 4 with TEAS or sham TEAS manipulation. The severity of illness is rated on a 7-point scale. The CGI-S includes seven options for scoring: 1 normal, not at all ill; 2 borderline mentally ill; 3 mildly ill; 4 moderately ill; 5 markedly ill; 6 severely ill; and 7 among the most extremely ill patients. Clinical response at week 4 was defined as a rating of “moderately ill” to “mildly ill” (4 or 3), which is considered a clinically meaningful response.

The other secondary outcomes included CPRS-R: S score, CPRS-R: S score, go/no-go performances, and the concentration of oxygenated hemoglobin (HbO) at CH 37 within frontal lobe cortex at week 4 and its changes from baseline to week 4. The CPRS-R:S is a teacher-rated 27-item 4-point Likert rating scale that contains items on ADHD and comorbid conditions. The CTRS-R:S is a teacher-rated 28-item 4-point Likert rating scale that contains items on ADHD and comorbid conditions. The accuracy (ACC) for go and no-go trials and reaction time (RT) of go trials were computed for each go/no-go block. The significant increase of the HbO concentration in the specific channel from baseline to week 4 was considered as an enhance of the regional cerebral blood flow.

4. Statistical Analysis

The sample size was calculated by using G*Power statistical software version 3.1.9.2 (University of Dusseldorf) according to apriori computation, where 36 participants are necessary for 0.8 statistical power to detect a 2-sided significance level of 5%, and 78 participants were used in case of potential sample missing.

Statistical analyses were based on the intention-to-treat population of randomly assigned patients, and were done with IBM SPSS Statistics 19. Baseline characteristics of groups TEAS and sham TEAS were described by mean (SD). Primary and secondary outcomes’ differences between the two

groups were analyzed by calculating the mean (95% CI). To analyze the differences in primary outcome measures between both randomized groups with the group from baseline to 4 weeks, we used a chi-square test on dichotomized CGI-I scores, comparing “very much improved” and “much improved” (coded as improved) with all else (coded as not improved). We used the group-by-time interaction of the mixed model for repeated measures (MMRM) to analyze differences in the other secondary outcomes between two groups, using group (TEAS or Sham TEAS) and time point (baseline or 4 weeks) as fixed effects. We added simple effects analysis to the MMRM analysis to investigate the inter-group and intra-group differences before and after the treatment between TEAS and Sham TEAS groups, so as to verify the treatment effect while ensuring the consistent experimental baseline. The significance threshold for all analyses was a P value of 0.05. All P values were adjusted by Bonferroni test.

5. Consent Documentation

All the subjects participated in the study voluntarily and signed a written informed consent. The study was approved by local ethic committee of human research at the First Affiliated Hospital of Xi’an Jiaotong University in China.
