# Supplementary material for: Transcutaneous electrical acupoint stimulation for children with attention-deficit/hyperactivity disorder: a randomized clinical trial
Source: Transl Psychiatry. 2022 Apr 21;12:165. doi: 10.1038/s41398-022-01914-0 (PMC9022403; doi:10.1038/s41398-022-01914-0)

**eTable 1. Baseline Characteristics of Patients in the True TEAS and Sham TEAS Groups in the PP Population**

| **Characteristics** | **TEAS (n=37)** | **Sham TEAS (n=31)** |
| --- | --- | --- |
| **Male, No. (%)** | 31 (83.8) | 25 (80.6) |
| **Age, mean (SD), year** | 8.1 (1.2) | 8.4 (1.4) |
| **IQ, mean (SD), score** | 94.0 (12.6) | 96.4 (11.0) |
| **BMI, mean (SD)** | 17.4 (2.8) | 17.2 (3.3) |
| **Subtype, No. (%)** |  |  |
| **ADHD-I** | 17 (45.9) | 15 (48.4) |
| **ADHD-HI** | 0 (0) | 0 (0) |
| **ADHD-C** | 20 (54.1) | 16 (51.6) |
| **Ethnicity, No. (%)** |  |  |
| **Han** | 37 (100) | 30 (96.8) |
| **Others** | 0 | 1 |

Abbreviations: TEAS, transcutaneous electrical acupoint stimulation; PP, planned per protocol; IQ, intelligence quotient; BMI, body mass index.

**eTable 2. Primary and Secondary Outcomes from PP Analysis**

|  | **TEAS (n=37)** | **Sham TEAS (n=31)** | ***P* value** | | | | |
| --- | --- | --- | --- | --- | --- | --- | --- |
| **Primary outcome** | | | | | | | |
| **CGI-I** | | | | | | | |
| **Score at wk 4^a^** | | | | | | | |
| **Improved, No. (%)**  **Not improved, No. (%)** | 13 (35.1)  24 (64.9) | 3 (9.7)  28 (90.3) | .014* | | | | |
| **Secondary outcomes** | | |  | **TEAS and Sham TEAS** | | **Baseline and Week 4** | |
|  |  |  |  | **Baseline** | **Week 4** | **TEAS** | **Sham TEAS** |
| **CGI-S, mean (95% CI)** | | | | | | | |
| **Score at baseline**  **Score at wk 4**  **Change at wk 4^b^** | 4.41 (4.16 to 4.65)  3.49 (3.20 to 3.78)  -0.92 (-1.21 to -0.63) | 4.65 (4.34 to 4.95)  4.35 (3.98 to 4.73)  -0.29 (-0.56 to -0.02) | .002* | .257 | < .001* | < .001* | .050 |
| **CPRS-R: S, mean (95% CI)** | | | | | | | |
| **Score at baseline**  **Score at wk 4**  **Change at wk 4^c^** | 45.78 (41.70 to 49.86)  38.92 (34.03 to 43.81)  -6.86 (-10.50 to -3.23) | 47.81 (44.50 to 51.12)  41.97 (36.80 to 47.14)  -5.84 (-9.62 to -2.06) | .693 | .517 | .330 | < .001* | .003* |
| **CTRS-R: S, mean (95% CI)** | | | | | | | |
| **Score at baseline**  **Score at wk 4**  **Change at wk 4^d^** | 31.19 (26.00 to 36.37)  29.05 (23.37 to 34.74)  -2.14 (-6.35 to 2.08) | 35.39 (30.16 to 40.61)  33.45 (27.28 to 39.62)  -1.94 (-6.67 to 2.79) | .949 | .284 | .262 | .312 | .401 |
| **ACC (%), mean (95% CI)** | | | | | | | |
| **At baseline**  **At wk 4**  **Change at wk 4^e^** | 84.17 (79.73 to 88.60)  91.44 (88.80 to 94.08)  7.27 (3.56 to 10.99) | 84.93 (81.60 to 88.25)  87.17 (84.24 to 90.11)  2.25 (-1.81 to 6.30) | .067 | .753 | .080 | < .001* | .264 |
| **RT (ms), mean (95% CI)** | | | | | | | |
| **At baseline**  **At wk 4**  **Change at wk 4^f^** | 305.9 (274.2 to 337.5)  369.9 (328.1 to 411.6)  64.0 (11.9 to 116.2) | 315.8 (276.4 to 355.2)  331.1 (262.8 to 399.3)  15.3 (-48.9 to 79.5) | .230 | .757 | .227 | .021* | .608 |
| **Oxy-HB CH 37 (mM mm), mean (95% CI)** | | | | | | | |
| **At baseline**  **At wk 4**  **Change at wk 4^g^** | 0.021 (-0.008 to 0.051)  0.126 (0.083 to 0.170)  0.105 (0.068 to 0.142) | 0.025 (0.005 to 0.044)  0.030 (-0.013 to 0.072)  0.005 (-0.049 to 0.058) | .002* | .891 | < .001* | < .001* | .837 |

^a^ Chi-square to test the CGI-I scores at week 4. ^b-g^ Indicates the difference in mean change from baseline to endpoint between the TEAS and sham TEAS groups by MMRM and the Wilcoxon rank-sum test. The main effect for time was signiﬁcant for the ^b^ CGI-S score (Z = -2.955, *P* = .003) and ^g^ Oxy-HB CH 37 (Z = -4.464, *P* < .001) but not for the ^c^ CPRS-R: S score (Z = -.520, *P* = .603), ^d^ CTRS-R: S score (Z = -.313, *P* = .754), ^e^ ACC (Z = -1.865, *P* = .062) and ^f^ RT (Z = -1.520, *P* = .129). Abbreviations: ITT, intention to treat; TEAS, transcutaneous electrical acupoint stimulation; CGI-I, Clinical Global Impression Scale-Improvement of Illness; CGI-S, Clinical Global Impression-Severity of Illness; CPRS-R: S, Conners’ Parent Rating Scales-Revised: Short Form; CTRS-R: S, Conners’ Teacher Rating Scales-Revised: Short Form; ACC, accuracy; RT, reaction time; Oxy-Hb, oxygenated hemoglobin; SD, standard deviation.

**eTable 3. Summary of Fixed Effect Results by Mixed Models for Repeated Measurements Analysis in the ITT Analysis**

| **Secondary outcomes** | ***P* value** | | |
| --- | --- | --- | --- |
|  | **TEAS and Sham TEAS** | **Baseline and Week 4** | **(Group-Time)** |
| **CGI-S** | .001* | < .001* | .001* |
| **CPRS-R: S** | .347 | < .001* | .384 |
| **CTRS-R: S** | .192 | .152 | .956 |
| **ACC** | .449 | < .001* | .049* |
| **RT** | .676 | .034* | .193 |
| **Oxy-HB CH37** | .012* | < .001* | .001* |

Abbreviations: TEAS, transcutaneous electrical acupoint stimulation; CGI-S, Clinical Global Impressions Scale - Severity of Illness; CPRS-R: S, Conners’ Parent Rating Scales-Revised: Short Form; CTRS-R: S, Conners’ Teacher Rating Scales-Revised: Short Form; ACC, accuracy; RT, reaction time; Oxy-Hb, oxygenated hemoglobin.

**eTable 4. Summary of Fixed Effect Results by Mixed Models for Repeated Measurements Analysis in the PP Analysis**

| **Secondary outcomes** | ***P* value** | | |
| --- | --- | --- | --- |
|  | **TEAS and Sham TEAS** | **Baseline and Week 4** | **(Group-Time)** |
| **CGI-S** | .004* | < .001* | .002* |
| **CPRS-R: S** | .373 | < .001* | .693 |
| **CTRS-R: S** | .233 | .195 | .949 |
| **ACC** | .385 | .001* | .067 |
| **RT** | .562 | .053 | .230 |
| **Oxy-HB CH37** | .020* | .001* | .002* |

Abbreviations: PP, planned per protocol; TEAS, transcutaneous electrical acupoint stimulation; CGI-S, Clinical Global Impressions Scale - Severity of Illness; CPRS-R: S, Conners’ Parent Rating Scales-Revised: Short Form; CTRS-R: S, Conners’ Teacher Rating Scales-Revised: Short Form; ACC, accuracy; RT, reaction time; Oxy-Hb, oxygenated hemoglobin.

**eFig. 1 The electrodes used in the present trial**


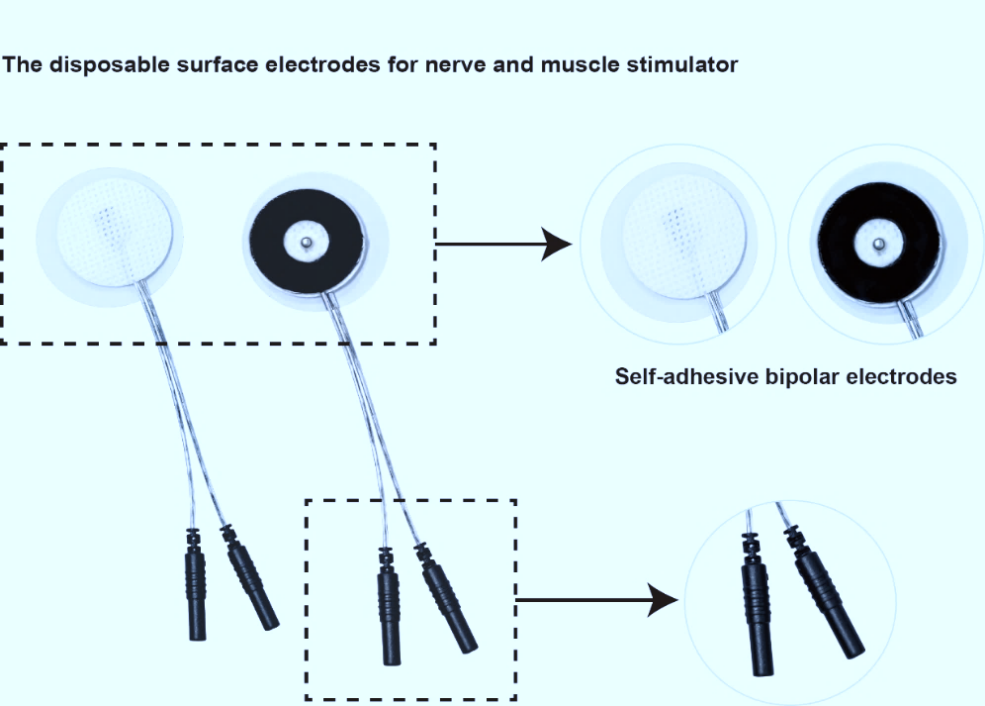

Supplement: Supplementary file 2 — Supplement 2 [file 41398_2022_1914_MOESM2_ESM.docx]
