# Supplementary material for: Transcutaneous electrical acupoint stimulation for children with attention-deficit/hyperactivity disorder: a randomized clinical trial
Source: Transl Psychiatry. 2022 Apr 21;12:165. doi: 10.1038/s41398-022-01914-0 (PMC9022403; doi:10.1038/s41398-022-01914-0)
Supplement: Supplementary file 3 — Supplement 3 [file 41398_2022_1914_MOESM3_ESM.pdf]

B-CH-1	B-CH-2	B-CH-3	B-CH-4	B-CH-5	B-CH-6	B-CH-7	B-CH-8	B-CH-9
-0.093	-0.017	-0.025	-0.001	-0.024	-0.016	-0.006	0.004	-0.075
-0.190	-0.094	-0.008	-0.023	-0.025	-0.021	-0.044	-0.023	-0.042
-0.035	0.002	-0.015	-0.017	0.058	0.055	0.017	-0.085	-0.043
-0.026	-0.014	0.054	0.049	0.195	0.071	0.048	-0.143	-0.033
0.024	0.012	0.037	0.025	0.012	-0.022	0.040	-0.048	0.022
-0.007	-0.026	-0.060	-0.060	-0.066	-0.041	-0.041	-0.064	-0.048
-0.023	0.005	-0.032	-0.004	0.020	0.013	-0.012	0.071	0.073
0.053	-0.010	-0.032	0.061	0.034	0.030	0.051	0.069	0.056
-0.064	0.013	0.017	-0.009	-0.038	0.023	-0.006	-0.048	-0.061
-0.059	-0.057	-0.071	-0.089	-0.053	-0.054	-0.058	-0.221	-0.154
-0.091	-0.103	-0.024	-0.037	-0.021	-0.034	-0.073	-0.148	-0.053
-0.071	-0.095	-0.073	-0.034	-0.023	-0.026	-0.015	-0.031	-0.054
-0.024	-0.023	-0.049	-0.015	-0.001	-0.022	-0.046	-0.035	-0.096
-0.104	-0.004	-0.043	-0.025	-0.031	-0.040	-0.040	-0.054	-0.032
-0.134	-0.069	-0.038	-0.034	-0.022	-0.095	-0.089	-0.068	-0.077
-0.031	0.052	0.068	-0.009	-0.056	0.017	0.034	0.132	0.011
0.048	0.049	0.084	0.039	0.021	0.010	0.057	0.049	0.116
-0.035	-0.127	-0.082	-0.033	-0.074	0.011	-0.066	-0.072	-0.063
-0.032	0.143	0.041	-0.012	0.004	0.020	0.064	-0.013	0.021
-0.006	0.058	0.023	0.028	0.019	0.025	0.025	0.047	0.009
-0.004	0.018	0.038	0.011	0.026	0.013	0.006	0.017	0.006
-0.036	0.011	0.067	0.055	0.002	-0.048	0.014	0.082	0.013
-0.262	-0.133	-0.308	-0.196	-0.331	-0.036	-0.229	-0.469	-0.232
-0.229	-0.036	-0.018	-0.054	-0.005	0.074	0.034	-0.125	-0.231
0.224	0.303	0.304	-0.142	0.013	-0.010	0.037	0.098	0.010
-0.311	0.285	0.072	0.021	-0.033	0.399	0.049	-0.396	0.239
-0.362	-0.216	-0.159	-0.208	-0.104	-0.013	-0.086	-0.271	-0.152
-0.065	0.147	-0.112	-0.090	-0.047	-0.206	-0.201	-0.204	-0.041
-0.389	-0.654	-0.376	-0.116	0.104	-0.346	-0.075	-0.162	-0.436
-0.021	-0.054	-0.020	0.232	0.123	0.042	-0.008	-0.089	-0.095
-0.262	-0.454	-0.286	-0.066	-0.007	0.047	-0.143	-0.280	-0.395
-0.280	-0.464	-0.409	-0.094	-0.115	-0.139	-0.362	-0.940	-0.133
0.188	-0.242	-0.073	-0.101	-0.072	0.297	0.215	-0.166	0.062
0.208	0.213	0.439	0.191	-0.178	-0.082	0.029	0.344	-0.007
0.885	0.917	0.872	0.756	0.748	0.519	0.642	0.391	0.900
0.573	-0.002	0.528	0.112	0.127	0.032	0.345	0.287	0.227
0.140	0.050	0.148	0.076	-0.117	0.030	0.057	0.096	0.063
0.012	-0.095	-0.223	0.145	-0.060	-0.432	-0.288	-0.547	-0.033
-0.571	0.296	-0.495	-0.250	0.210	-0.110	-0.211	0.031	0.006

B-CH-10	B-CH-11	B-CH-12	B-CH-13	B-CH-14	B-CH-15	B-CH-16	B-CH-17	B-CH-18
-0.059	-0.001	-0.016	-0.048	-0.032	-0.005	0.000	-0.001	-0.015
-0.052	-0.131	-0.019	-0.009	-0.045	-0.030	-0.038	-0.037	-0.037
-0.055	-0.088	-0.065	-0.006	0.027	0.035	0.087	0.046	-0.027
-0.099	-0.012	-0.039	0.056	-0.010	0.082	0.009	0.027	0.048
0.099	0.040	-0.068	0.037	0.113	0.048	-0.048	0.019	0.055
-0.033	0.009	0.017	-0.035	-0.033	-0.074	-0.054	-0.057	-0.047
0.007	-0.002	-0.032	0.022	0.027	0.003	-0.030	-0.017	-0.016
0.045	0.046	0.008	-0.036	0.037	0.045	0.049	0.062	0.029
-0.096	-0.042	0.043	0.030	0.016	-0.118	-0.018	0.013	0.021
-0.090	-0.063	0.018	-0.120	0.011	-0.054	-0.039	-0.032	-0.042
-0.076	-0.085	-0.117	-0.050	-0.034	-0.044	-0.054	-0.058	-0.062
-0.041	-0.032	-0.034	-0.075	-0.047	-0.041	-0.030	-0.038	-0.045
-0.132	-0.092	0.015	-0.019	-0.028	-0.021	-0.010	-0.017	-0.057
-0.221	-0.183	-0.012	-0.063	-0.054	-0.016	-0.037	-0.028	-0.021
-0.066	-0.111	-0.092	-0.065	-0.052	-0.032	-0.027	-0.020	-0.067
-0.044	-0.012	-0.009	0.040	0.061	0.003	-0.017	0.073	0.039
0.134	0.109	0.057	0.071	0.088	0.025	0.010	0.077	0.022
-0.093	-0.024	-0.105	-0.161	-0.040	-0.038	0.025	-0.033	-0.055
0.047	0.046	0.004	0.032	0.035	0.018	0.047	-0.014	0.026
0.004	0.006	0.011	0.011	0.028	0.008	0.010	0.032	0.042
0.011	-0.024	0.015	0.023	0.019	0.031	0.005	0.007	0.016
0.006	-0.015	-0.003	0.008	0.036	0.035	-0.025	-0.020	0.026
-0.275	-0.070	-0.060	-0.039	-0.173	-0.233	-0.044	-0.254	-0.117
-0.176	-0.502	0.214	0.017	-0.061	-0.178	0.141	0.066	0.038
-0.165	-0.049	0.291	0.240	-0.174	-0.108	0.000	-0.090	-0.081
-0.504	-0.119	0.223	-0.199	-0.111	0.115	0.186	0.503	-0.003
-0.446	-0.288	-0.310	-0.007	-0.046	-0.126	-0.078	-0.037	-0.079
-0.042	-0.172	-0.167	-0.108	-0.110	-0.021	-0.127	-0.172	-0.086
-0.180	-0.248	-0.581	-0.118	-0.181	0.085	-0.129	-0.010	-0.040
-0.019	-0.138	0.055	-0.116	0.004	0.169	-0.089	0.083	-0.078
-0.437	-0.051	-0.019	-0.319	-0.114	0.166	0.016	-0.102	0.008
-0.135	-0.098	-0.230	-0.148	-0.072	-0.014	-0.163	-0.248	-0.193
-0.052	0.034	0.225	-0.204	0.025	0.188	-0.434	-0.238	0.082
0.075	0.083	0.043	0.203	0.323	0.027	-0.185	-0.057	-0.021
0.899	0.726	0.439	0.217	0.385	0.602	0.294	0.466	0.269
-0.155	-0.184	0.212	0.379	0.242	0.104	0.172	0.258	0.221
0.132	0.047	-0.019	-0.016	0.097	0.055	-0.006	0.078	0.112
-0.168	0.214	-0.092	-0.059	0.087	-0.080	-0.208	-0.217	-0.321
0.617	-0.305	0.070	-0.036	-0.140	-0.197	0.030	-0.277	0.112

B-CH-19	B-CH-20	B-CH-21	B-CH-22	B-CH-23	B-CH-24	B-CH-25	B-CH-26	B-CH-27
-0.038	0.006	-0.025	0.004	-0.034	-0.041	-0.020	-0.008	0.027
0.007	0.072	-0.045	0.048	0.083	-0.082	-0.080	-0.029	-0.045
-0.014	-0.003	-0.047	-0.044	-0.037	0.009	0.074	0.007	0.014
-0.034	-0.049	-0.059	-0.044	0.052	0.041	0.051	-0.001	0.034
-0.039	0.032	0.044	0.004	0.040	0.054	0.141	0.023	-0.048
-0.092	-0.014	0.000	-0.019	-0.033	-0.020	-0.082	-0.094	-0.085
0.061	-0.028	0.011	-0.026	-0.033	0.004	0.008	-0.025	-0.031
0.036	0.036	0.025	0.019	0.032	0.032	0.038	0.062	0.065
-0.029	0.003	-0.046	0.031	0.089	-0.008	-0.043	-0.001	-0.002
-0.081	-0.021	-0.072	-0.013	-0.009	0.039	0.040	0.001	-0.015
-0.097	-0.073	-0.082	-0.100	-0.111	-0.023	-0.052	-0.036	-0.029
-0.042	-0.054	-0.002	-0.067	-0.048	-0.022	-0.047	-0.046	-0.042
-0.046	-0.079	-0.050	-0.021	0.009	-0.001	-0.024	-0.013	-0.004
-0.040	-0.091	-0.095	-0.064	-0.033	-0.078	-0.042	-0.008	-0.025
0.018	-0.071	-0.191	-0.144	-0.053	-0.058	-0.068	-0.028	0.121
0.040	-0.040	-0.029	-0.039	-0.015	0.079	0.054	0.022	-0.005
0.048	0.069	0.098	0.039	0.070	0.067	0.088	0.009	0.055
-0.036	-0.026	-0.168	-0.069	-0.054	-0.053	-0.039	-0.003	-0.034
0.020	0.026	0.071	-0.007	-0.001	0.004	0.033	0.021	0.027
0.022	-0.002	-0.024	0.006	0.018	0.040	0.036	0.013	0.011
0.022	0.028	0.013	0.024	0.027	0.015	0.043	0.007	0.015
0.023	-0.032	-0.023	-0.045	-0.006	-0.042	0.022	0.023	-0.012
-0.298	-0.538	-0.248	-0.057	0.032	-0.114	0.035	-0.321	-0.133
0.000	0.124	0.189	-0.010	-0.090	0.014	-0.157	0.060	0.261
0.182	0.015	-0.320	0.226	0.299	0.023	-0.135	0.020	-0.396
0.069	0.001	-0.762	0.015	0.024	-0.080	0.131	0.195	-0.101
-0.008	-0.050	-0.149	-0.049	-0.056	-0.022	-0.073	-0.071	-0.020
-0.110	-0.130	-0.005	1.031	0.200	-0.149	-0.049	-0.087	-0.161
-0.121	-0.413	-0.116	-0.326	-0.349	-0.158	0.017	-0.155	-0.103
-0.034	-0.055	0.055	-0.093	-0.012	0.022	0.086	-0.020	-0.002
-0.528	-0.198	-0.402	-0.027	0.379	-0.292	-0.289	0.130	0.002
-0.277	-0.084	-0.317	-0.225	0.047	-0.099	0.078	-0.227	-0.108
0.012	0.118	0.049	0.019	-0.122	-0.121	-0.095	-0.122	-0.113
0.143	-0.004	0.254	-0.188	-0.508	0.071	0.161	0.057	-0.065
0.597	0.582	0.364	0.498	0.599	0.388	0.573	0.239	0.239
0.122	0.104	0.438	-0.025	0.108	0.214	0.336	0.245	0.113
0.017	0.029	0.122	-0.038	0.001	-0.022	-0.100	-0.081	0.034
-0.196	0.006	-0.045	-0.019	0.022	0.068	0.045	0.031	0.141
-0.355	0.232	0.225	-0.397	0.155	-0.101	-0.138	0.027	-0.312

B-CH-28	B-CH-29	B-CH-30	B-CH-31	B-CH-32	B-CH-33	B-CH-34	B-CH-35	B-CH-36
0.024	-0.033	0.002	0.006	-0.005	-0.019	-0.004	-0.029	-0.027
-0.017	-0.028	0.113	0.065	0.042	0.168	0.092	-0.040	-0.032
0.027	-0.001	0.007	-0.040	-0.015	-0.017	-0.035	0.049	0.050
-0.024	-0.048	0.025	-0.035	-0.002	-0.014	0.032	0.006	0.075
0.077	-0.038	0.002	0.049	0.043	0.014	0.014	0.124	0.091
-0.056	-0.110	-0.136	-0.043	-0.018	-0.024	0.015	-0.041	-0.103
0.005	0.085	0.049	0.022	0.007	-0.036	0.002	0.019	0.029
0.041	0.048	0.032	0.041	0.040	0.006	0.028	0.029	0.033
0.019	0.003	0.036	-0.036	0.014	0.070	0.053	0.035	0.027
-0.016	-0.081	-0.073	-0.066	0.017	0.041	0.047	0.095	-0.052
-0.041	-0.091	-0.090	-0.079	-0.057	-0.040	0.004	-0.048	-0.039
-0.048	-0.060	-0.016	-0.044	-0.006	0.005	0.024	-0.015	-0.055
-0.024	-0.059	-0.047	-0.044	-0.049	-0.014	-0.002	-0.004	-0.029
0.022	-0.086	-0.044	-0.099	-0.066	-0.025	-0.111	-0.097	-0.120
-0.035	-0.105	-0.026	-0.032	-0.005	-0.016	-0.026	-0.080	-0.136
0.056	0.083	-0.015	0.017	0.011	0.009	0.002	0.027	-0.015
0.116	0.055	0.044	0.032	-0.019	0.051	0.096	0.054	0.075
-0.010	-0.051	-0.058	-0.136	-0.055	0.035	0.024	-0.053	-0.032
0.009	0.011	0.006	0.025	0.063	-0.008	-0.059	0.024	0.007
0.022	0.061	0.015	0.023	-0.002	0.021	0.041	0.007	0.012
0.029	0.048	0.018	0.025	0.029	0.051	0.014	0.036	0.019
0.056	-0.024	-0.025	-0.048	-0.011	-0.009	-0.010	-0.013	-0.053
-0.348	-0.143	-0.172	-0.410	-0.170	0.023	0.107	-0.160	-0.361
0.059	0.193	0.300	0.504	-0.218	0.029	-0.061	-0.089	-0.213
0.011	-0.033	0.010	-0.014	0.101	0.275	0.223	-0.012	0.011
0.310	-0.084	0.156	-0.010	-0.246	-0.091	-0.001	-0.126	0.029
-0.035	-0.138	-0.013	0.196	-0.068	0.073	0.061	-0.066	-0.072
-0.124	-0.111	-0.055	-0.087	0.046	-0.016	-0.111	-0.110	-0.165
-0.018	-0.027	-0.219	-0.136	0.016	0.287	0.063	-0.014	-0.183
0.089	-0.143	0.040	-0.022	0.031	0.110	0.173	0.007	0.026
0.042	-0.214	-0.484	-0.678	0.008	0.326	0.258	-0.575	0.059
-0.084	-0.214	-0.062	-0.020	-0.315	0.035	-0.179	-0.343	-0.166
-0.150	-0.105	-0.061	0.173	0.112	0.060	-0.175	-0.152	-0.339
0.002	0.014	-0.078	0.215	-0.286	-0.841	-0.335	0.122	-0.019
0.372	0.534	0.936	1.343	0.272	0.877	0.155	0.448	0.480
0.173	0.354	0.202	0.221	0.405	0.006	0.117	0.299	0.192
-0.067	0.154	0.041	0.066	0.007	-0.078	-0.040	-0.040	0.113
0.220	0.051	-0.099	0.042	-0.115	-0.038	0.056	0.243	0.294
0.048	0.060	-0.107	-0.023	-0.056	0.039	0.090	-0.571	-0.387

B-CH-37	B-CH-38	B-CH-39	B-CH-40	B-CH-41	B-CH-42	B-CH-43	B-CH-44	B-CH-45
-0.056	0.036	0.002	0.013	0.017	0.011	-0.034	0.013	0.019
0.067	-0.036	-0.046	0.042	0.084	-0.052	0.037	0.048	-0.048
0.025	0.075	0.000	0.022	-0.004	-0.001	0.003	-0.010	-0.006
0.015	0.066	0.056	-0.052	0.033	-0.029	-0.012	-0.026	0.018
0.065	0.036	-0.057	0.001	0.037	0.051	0.081	-0.007	0.096
0.053	-0.073	-0.065	-0.033	-0.055	-0.016	-0.029	0.051	-0.041
-0.087	-0.003	0.013	0.053	0.040	0.038	0.027	0.012	0.037
-0.010	0.046	0.037	0.021	0.039	0.040	0.052	0.049	0.012
0.009	0.046	0.043	0.037	0.024	-0.069	0.015	0.049	0.048
-0.019	0.083	0.031	-0.044	-0.065	0.119	0.067	0.016	0.007
-0.014	-0.059	-0.072	-0.058	-0.076	-0.059	-0.014	0.007	-0.041
-0.007	-0.060	-0.056	0.000	0.021	-0.075	0.043	0.070	0.052
0.012	0.014	-0.047	-0.026	-0.023	0.040	-0.038	-0.045	-0.011
0.019	-0.037	-0.094	-0.041	-0.057	-0.121	-0.076	-0.032	-0.121
0.455	-0.060	-0.121	0.023	0.047	-0.045	-0.160	0.046	-0.027
-0.028	0.006	0.090	0.033	0.002	0.009	0.043	-0.018	-0.008
0.019	0.078	0.059	0.039	-0.010	0.029	0.007	0.073	0.054
0.034	-0.054	-0.016	-0.001	-0.026	-0.069	-0.015	0.028	-0.031
0.086	-0.056	0.041	0.021	0.014	-0.001	0.041	-0.036	0.044
-0.052	0.016	0.026	0.038	0.022	0.035	0.030	0.044	0.007
0.028	0.037	0.032	0.010	0.004	0.024	0.019	0.052	0.055
0.071	0.022	0.010	-0.003	-0.052	-0.003	-0.050	-0.005	-0.007
0.027	-0.744	-0.253	-0.169	0.097	-0.066	-0.057	0.041	0.158
0.012	-0.020	0.008	0.283	0.059	-0.025	-0.213	-0.349	0.081
0.118	-0.025	-0.034	0.020	0.076	0.042	0.162	0.093	0.128
-0.003	-0.147	-0.045	-0.061	0.518	-0.116	-0.229	-0.045	-0.118
-0.073	-0.003	0.000	0.197	0.279	0.174	0.169	0.049	-0.078
-0.098	-0.225	-0.189	0.127	0.162	0.056	0.061	0.010	-0.162
0.082	-0.110	0.147	0.132	-0.092	0.055	0.095	-0.054	-0.023
0.042	-0.007	-0.103	-0.042	0.286	0.090	0.191	0.464	0.145
-0.003	-0.032	-0.590	-0.613	-0.109	-0.277	0.058	0.596	0.182
-0.097	0.334	-0.062	-0.429	-0.019	-0.180	0.010	-0.167	-0.120
0.042	-0.761	-0.131	-0.234	-0.028	0.082	0.204	-0.202	-0.053
0.057	-0.103	0.008	-0.392	-0.161	-0.158	-0.628	-0.729	-0.249
0.011	0.312	0.174	0.202	0.409	1.293	0.274	0.390	0.122
0.012	0.065	0.144	0.150	0.255	0.243	0.513	0.381	0.344
0.010	0.148	-0.103	0.089	-0.032	0.181	-0.085	-0.174	0.047
-0.005	0.555	0.095	-0.043	0.200	0.104	-0.112	-0.063	0.247
-0.003	-0.364	-0.393	-0.058	0.162	0.347	-0.140	-0.050	-0.112

B-CH-46	B-CH-47	B-CH-48	B-CH-49	B-CH-50	B-CH-51	B-CH-52	A-CH-1
-0.043	-0.018	0.004	0.002	0.013	0.041	0.025	-0.163
-0.062	-0.037	0.004	-0.058	-0.064	-0.006	-0.011	0.039
0.077	0.032	0.014	0.050	-0.006	0.038	0.019	0.039
0.125	0.099	0.019	0.010	-0.009	0.041	0.017	0.012
0.083	0.006	-0.007	-0.060	-0.043	-0.030	0.011	-0.138
-0.084	-0.076	-0.099	-0.077	-0.041	0.006	-0.006	-0.262
0.026	-0.009	0.008	0.032	0.005	0.040	0.054	-0.229
0.074	0.049	0.083	0.089	0.034	0.045	0.049	0.224
-0.011	0.012	0.019	0.034	0.075	0.152	0.055	-0.311
0.007	-0.001	-0.033	0.067	0.023	0.036	0.029	-0.362
-0.071	-0.044	-0.047	-0.093	-0.081	-0.009	-0.073	-0.065
-0.055	-0.025	-0.066	-0.036	-0.039	0.051	0.010	-0.389
-0.012	-0.011	-0.019	-0.042	-0.040	-0.049	-0.014	-0.021
-0.050	-0.020	0.005	-0.075	-0.034	-0.014	-0.037	-0.262
-0.092	-0.156	-0.241	-0.075	-0.052	0.193	0.081	-0.280
-0.012	0.029	-0.035	0.055	0.033	-0.029	0.005	0.188
0.077	0.039	0.075	0.075	0.045	0.001	-0.005	0.208
-0.028	-0.020	-0.029	-0.038	0.034	0.034	-0.027	0.885
0.100	-0.112	-0.135	-0.029	0.036	0.051	0.014	0.573
0.022	0.018	-0.004	0.017	0.057	0.047	0.033	0.140
0.019	0.013	0.031	0.018	0.013	0.009	0.009	0.012
-0.035	-0.032	0.035	-0.005	-0.024	0.004	0.017	-0.571
-0.303	-0.310	-0.278	-0.359	-0.287	0.171	0.116	-0.007
0.061	0.108	-0.043	0.041	0.158	0.087	0.274	-0.023
0.013	0.009	0.208	0.071	0.026	0.074	0.228	0.053
-0.140	-0.140	-0.119	0.058	-0.192	0.259	0.296	-0.064
-0.045	-0.099	0.014	0.019	-0.016	0.474	0.232	-0.059
-0.151	-0.136	-0.151	-0.179	0.057	0.159	0.064	-0.091
0.087	-0.268	0.017	0.362	0.144	0.045	-0.074	-0.071
0.019	-0.084	-0.090	-0.066	-0.072	0.505	0.211	-0.024
-0.268	-0.057	-0.035	-0.350	-0.920	0.131	0.068	-0.104
-0.094	-0.317	0.124	-0.317	-0.254	0.029	0.096	-0.134
-0.192	-0.136	-0.107	-0.600	-0.232	-0.130	0.093	-0.031
0.086	-0.018	-0.050	-0.007	-0.174	-0.949	-0.628	0.048
0.229	0.076	0.212	0.403	0.064	-0.029	1.094	-0.035
0.219	0.292	0.129	0.164	0.061	-0.034	0.303	-0.032
0.189	0.122	0.021	0.142	0.133	0.036	0.031	-0.006
0.133	0.095	0.216	0.165	0.022	0.057	0.083	-0.004
-0.291	-0.457	-0.547	-0.555	-0.138	0.034	0.264	-0.036

A-CH-2	A-CH-3	A-CH-4	A-CH-5	A-CH-6	A-CH-7	A-CH-8	A-CH-9	A-CH-10
-0.152	-0.024	-0.002	0.058	0.122	0.083	-0.059	0.029	0.115
0.044	-0.175	0.002	-0.052	0.002	0.167	0.006	0.009	-0.100
-0.126	-0.087	-0.045	0.084	-0.006	-0.014	0.060	0.023	-0.032
-0.115	0.062	0.127	-0.166	-0.270	0.381	0.070	0.216	0.013
-0.261	0.393	0.387	0.125	-0.038	0.029	0.061	-0.318	-0.077
-0.133	-0.308	-0.196	-0.331	-0.036	-0.229	-0.469	-0.232	-0.275
-0.036	-0.018	-0.054	-0.005	0.074	0.034	-0.125	-0.231	-0.176
0.303	0.304	-0.142	0.013	-0.010	0.037	0.098	0.010	-0.165
0.285	0.072	0.021	-0.033	0.399	0.049	-0.396	0.239	-0.504
-0.216	-0.159	-0.208	-0.104	-0.013	-0.086	-0.271	-0.152	-0.446
0.147	-0.112	-0.090	-0.047	-0.206	-0.201	-0.204	-0.041	-0.042
-0.654	-0.376	-0.116	0.104	-0.346	-0.075	-0.162	-0.436	-0.180
-0.054	-0.020	0.232	0.123	0.042	-0.008	-0.089	-0.095	-0.019
-0.454	-0.286	-0.066	-0.007	0.047	-0.143	-0.280	-0.395	-0.437
-0.464	-0.409	-0.094	-0.115	-0.139	-0.362	-0.940	-0.133	-0.135
-0.242	-0.073	-0.101	-0.072	0.297	0.215	-0.166	0.062	-0.052
0.213	0.439	0.191	-0.178	-0.082	0.029	0.344	-0.007	0.075
0.917	0.872	0.756	0.748	0.519	0.642	0.391	0.900	0.899
-0.002	0.528	0.112	0.127	0.032	0.345	0.287	0.227	-0.155
0.050	0.148	0.076	-0.117	0.030	0.057	0.096	0.063	0.132
-0.095	-0.223	0.145	-0.060	-0.432	-0.288	-0.547	-0.033	-0.168
0.296	-0.495	-0.250	0.210	-0.110	-0.211	0.031	0.006	0.617
-0.026	-0.060	-0.060	-0.066	-0.041	-0.041	-0.064	-0.048	-0.033
0.005	-0.032	-0.004	0.020	0.013	-0.012	0.071	0.073	0.007
-0.010	-0.032	0.061	0.034	0.030	0.051	0.069	0.056	0.045
0.013	0.017	-0.009	-0.038	0.023	-0.006	-0.048	-0.061	-0.096
-0.057	-0.071	-0.089	-0.053	-0.054	-0.058	-0.221	-0.154	-0.090
-0.103	-0.024	-0.037	-0.021	-0.034	-0.073	-0.148	-0.053	-0.076
-0.095	-0.073	-0.034	-0.023	-0.026	-0.015	-0.031	-0.054	-0.041
-0.023	-0.049	-0.015	-0.001	-0.022	-0.046	-0.035	-0.096	-0.132
-0.004	-0.043	-0.025	-0.031	-0.040	-0.040	-0.054	-0.032	-0.221
-0.069	-0.038	-0.034	-0.022	-0.095	-0.089	-0.068	-0.077	-0.066
0.052	0.068	-0.009	-0.056	0.017	0.034	0.132	0.011	-0.044
0.049	0.084	0.039	0.021	0.010	0.057	0.049	0.116	0.134
-0.127	-0.082	-0.033	-0.074	0.011	-0.066	-0.072	-0.063	-0.093
0.143	0.041	-0.012	0.004	0.020	0.064	-0.013	0.021	0.047
0.058	0.023	0.028	0.019	0.025	0.025	0.047	0.009	0.004
0.018	0.038	0.011	0.026	0.013	0.006	0.017	0.006	0.011
0.011	0.067	0.055	0.002	-0.048	0.014	0.082	0.013	0.006

A-CH-11	A-CH-12	A-CH-13	A-CH-14	A-CH-15	A-CH-16	A-CH-17	A-CH-18	A-CH-19
-0.158	-0.085	0.010	0.055	0.040	0.061	0.210	0.013	-0.065
0.091	0.073	-0.121	-0.093	-0.050	-0.035	0.095	0.071	0.020
-0.258	0.096	-0.060	-0.011	-0.037	0.007	0.032	0.019	-0.047
0.570	-0.128	0.168	0.056	0.182	-0.102	-0.297	0.397	-0.287
-0.272	0.196	0.100	0.276	0.168	-0.082	-0.195	0.044	-0.633
-0.070	-0.060	-0.039	-0.173	-0.233	-0.044	-0.254	-0.117	-0.298
-0.502	0.214	0.017	-0.061	-0.178	0.141	0.066	0.038	0.000
-0.049	0.291	0.240	-0.174	-0.108	0.000	-0.090	-0.081	0.182
-0.119	0.223	-0.199	-0.111	0.115	0.186	0.503	-0.003	0.069
-0.288	-0.310	-0.007	-0.046	-0.126	-0.078	-0.037	-0.079	-0.008
-0.172	-0.167	-0.108	-0.110	-0.021	-0.127	-0.172	-0.086	-0.110
-0.248	-0.581	-0.118	-0.181	0.085	-0.129	-0.010	-0.040	-0.121
-0.138	0.055	-0.116	0.004	0.169	-0.089	0.083	-0.078	-0.034
-0.051	-0.019	-0.319	-0.114	0.166	0.016	-0.102	0.008	-0.528
-0.098	-0.230	-0.148	-0.072	-0.014	-0.163	-0.248	-0.193	-0.277
0.034	0.225	-0.204	0.025	0.188	-0.434	-0.238	0.082	0.012
0.083	0.043	0.203	0.323	0.027	-0.185	-0.057	-0.021	0.143
0.726	0.439	0.217	0.385	0.602	0.294	0.466	0.269	0.597
-0.184	0.212	0.379	0.242	0.104	0.172	0.258	0.221	0.122
0.047	-0.019	-0.016	0.097	0.055	-0.006	0.078	0.112	0.017
0.214	-0.092	-0.059	0.087	-0.080	-0.208	-0.217	-0.321	-0.196
-0.305	0.070	-0.036	-0.140	-0.197	0.030	-0.277	0.112	-0.355
0.009	0.017	-0.035	-0.033	-0.074	-0.054	-0.057	-0.047	-0.092
-0.002	-0.032	0.022	0.027	0.003	-0.030	-0.017	-0.016	0.061
0.046	0.008	-0.036	0.037	0.045	0.049	0.062	0.029	0.036
-0.042	0.043	0.030	0.016	-0.118	-0.018	0.013	0.021	-0.029
-0.063	0.018	-0.120	0.011	-0.054	-0.039	-0.032	-0.042	-0.081
-0.085	-0.117	-0.050	-0.034	-0.044	-0.054	-0.058	-0.062	-0.097
-0.032	-0.034	-0.075	-0.047	-0.041	-0.030	-0.038	-0.045	-0.042
-0.092	0.015	-0.019	-0.028	-0.021	-0.010	-0.017	-0.057	-0.046
-0.183	-0.012	-0.063	-0.054	-0.016	-0.037	-0.028	-0.021	-0.040
-0.111	-0.092	-0.065	-0.052	-0.032	-0.027	-0.020	-0.067	0.018
-0.012	-0.009	0.040	0.061	0.003	-0.017	0.073	0.039	0.040
0.109	0.057	0.071	0.088	0.025	0.010	0.077	0.022	0.048
-0.024	-0.105	-0.161	-0.040	-0.038	0.025	-0.033	-0.055	-0.036
0.046	0.004	0.032	0.035	0.018	0.047	-0.014	0.026	0.020
0.006	0.011	0.011	0.028	0.008	0.010	0.032	0.042	0.022
-0.024	0.015	0.023	0.019	0.031	0.005	0.007	0.016	0.022
-0.015	-0.003	0.008	0.036	0.035	-0.025	-0.020	0.026	0.023

A-CH-20	A-CH-21	A-CH-22	A-CH-23	A-CH-24	A-CH-25	A-CH-26	A-CH-27	A-CH-28
-0.124	0.221	-0.139	-0.004	-0.010	0.003	-0.030	0.157	0.127
0.180	0.111	0.109	-0.126	-0.024	0.077	0.002	-0.028	0.006
0.127	-0.120	0.213	0.068	0.066	0.119	0.037	0.045	0.122
0.005	0.167	0.120	-0.073	0.027	0.134	-0.173	-0.120	0.236
0.146	-0.051	0.315	0.321	0.175	0.373	0.104	-0.033	-0.131
-0.538	-0.248	-0.057	0.032	-0.114	0.035	-0.321	-0.133	-0.348
0.124	0.189	-0.010	-0.090	0.014	-0.157	0.060	0.261	0.059
0.015	-0.320	0.226	0.299	0.023	-0.135	0.020	-0.396	0.011
0.001	-0.762	0.015	0.024	-0.080	0.131	0.195	-0.101	0.310
-0.050	-0.149	-0.049	-0.056	-0.022	-0.073	-0.071	-0.020	-0.035
-0.130	-0.005	1.031	0.200	-0.149	-0.049	-0.087	-0.161	-0.124
-0.413	-0.116	-0.326	-0.349	-0.158	0.017	-0.155	-0.103	-0.018
-0.055	0.055	-0.093	-0.012	0.022	0.086	-0.020	-0.002	0.089
-0.198	-0.402	-0.027	0.379	-0.292	-0.289	0.130	0.002	0.042
-0.084	-0.317	-0.225	0.047	-0.099	0.078	-0.227	-0.108	-0.084
0.118	0.049	0.019	-0.122	-0.121	-0.095	-0.122	-0.113	-0.150
-0.004	0.254	-0.188	-0.508	0.071	0.161	0.057	-0.065	0.002
0.582	0.364	0.498	0.599	0.388	0.573	0.239	0.239	0.372
0.104	0.438	-0.025	0.108	0.214	0.336	0.245	0.113	0.173
0.029	0.122	-0.038	0.001	-0.022	-0.100	-0.081	0.034	-0.067
0.006	-0.045	-0.019	0.022	0.068	0.045	0.031	0.141	0.220
0.232	0.225	-0.397	0.155	-0.101	-0.138	0.027	-0.312	0.048
-0.014	0.000	-0.019	-0.033	-0.020	-0.082	-0.094	-0.085	-0.056
-0.028	0.011	-0.026	-0.033	0.004	0.008	-0.025	-0.031	0.005
0.036	0.025	0.019	0.032	0.032	0.038	0.062	0.065	0.041
0.003	-0.046	0.031	0.089	-0.008	-0.043	-0.001	-0.002	0.019
-0.021	-0.072	-0.013	-0.009	0.039	0.040	0.001	-0.015	-0.016
-0.073	-0.082	-0.100	-0.111	-0.023	-0.052	-0.036	-0.029	-0.041
-0.054	-0.002	-0.067	-0.048	-0.022	-0.047	-0.046	-0.042	-0.048
-0.079	-0.050	-0.021	0.009	-0.001	-0.024	-0.013	-0.004	-0.024
-0.091	-0.095	-0.064	-0.033	-0.078	-0.042	-0.008	-0.025	0.022
-0.071	-0.191	-0.144	-0.053	-0.058	-0.068	-0.028	0.121	-0.035
-0.040	-0.029	-0.039	-0.015	0.079	0.054	0.022	-0.005	0.056
0.069	0.098	0.039	0.070	0.067	0.088	0.009	0.055	0.116
-0.026	-0.168	-0.069	-0.054	-0.053	-0.039	-0.003	-0.034	-0.010
0.026	0.071	-0.007	-0.001	0.004	0.033	0.021	0.027	0.009
-0.002	-0.024	0.006	0.018	0.040	0.036	0.013	0.011	0.022
0.028	0.013	0.024	0.027	0.015	0.043	0.007	0.015	0.029
-0.032	-0.023	-0.045	-0.006	-0.042	0.022	0.023	-0.012	0.056

A-CH-29	A-CH-30	A-CH-31	A-CH-32	A-CH-33	A-CH-34	A-CH-35	A-CH-36	A-CH-37
0.158	0.142	0.101	-0.083	-0.162	-0.149	-0.028	-0.128	-0.031
0.059	0.105	0.347	0.091	0.249	0.032	-0.007	0.000	-0.060
-0.010	0.000	0.035	0.108	0.176	0.171	0.170	0.008	0.106
-0.098	0.017	-0.004	0.194	-0.079	-0.195	-0.069	0.019	0.133
-0.228	-0.504	-0.134	0.091	0.207	0.054	0.331	0.495	0.079
-0.143	-0.172	-0.410	-0.170	0.023	0.107	-0.160	-0.361	0.032
0.193	0.300	0.504	-0.218	0.029	-0.061	-0.089	-0.213	0.119
-0.033	0.010	-0.014	0.101	0.275	0.223	-0.012	0.011	-0.001
-0.084	0.156	-0.010	-0.246	-0.091	-0.001	-0.126	0.029	0.368
-0.138	-0.013	0.196	-0.068	0.073	0.061	-0.066	-0.072	-0.018
-0.111	-0.055	-0.087	0.046	-0.016	-0.111	-0.110	-0.165	-0.001
-0.027	-0.219	-0.136	0.016	0.287	0.063	-0.014	-0.183	-0.006
-0.143	0.040	-0.022	0.031	0.110	0.173	0.007	0.026	0.014
-0.214	-0.484	-0.678	0.008	0.326	0.258	-0.575	0.059	0.020
-0.214	-0.062	-0.020	-0.315	0.035	-0.179	-0.343	-0.166	0.624
-0.105	-0.061	0.173	0.112	0.060	-0.175	-0.152	-0.339	0.042
0.014	-0.078	0.215	-0.286	-0.841	-0.335	0.122	-0.019	0.133
0.534	0.936	1.343	0.272	0.877	0.155	0.448	0.480	0.262
0.354	0.202	0.221	0.405	0.006	0.117	0.299	0.192	0.138
0.154	0.041	0.066	0.007	-0.078	-0.040	-0.040	0.113	0.097
0.051	-0.099	0.042	-0.115	-0.038	0.056	0.243	0.294	0.050
0.060	-0.107	-0.023	-0.056	0.039	0.090	-0.571	-0.387	0.147
-0.110	-0.136	-0.043	-0.018	-0.024	0.015	-0.041	-0.103	0.149
0.085	0.049	0.022	0.007	-0.036	0.002	0.019	0.029	0.189
0.048	0.032	0.041	0.040	0.006	0.028	0.029	0.033	0.211
0.003	0.036	-0.036	0.014	0.070	0.053	0.035	0.027	0.021
-0.081	-0.073	-0.066	0.017	0.041	0.047	0.095	-0.052	0.171
-0.091	-0.090	-0.079	-0.057	-0.040	0.004	-0.048	-0.039	0.240
-0.060	-0.016	-0.044	-0.006	0.005	0.024	-0.015	-0.055	0.132
-0.059	-0.047	-0.044	-0.049	-0.014	-0.002	-0.004	-0.029	0.093
-0.086	-0.044	-0.099	-0.066	-0.025	-0.111	-0.097	-0.120	0.140
-0.105	-0.026	-0.032	-0.005	-0.016	-0.026	-0.080	-0.136	0.146
0.083	-0.015	0.017	0.011	0.009	0.002	0.027	-0.015	0.384
0.055	0.044	0.032	-0.019	0.051	0.096	0.054	0.075	0.113
-0.051	-0.058	-0.136	-0.055	0.035	0.024	-0.053	-0.032	0.011
0.011	0.006	0.025	0.063	-0.008	-0.059	0.024	0.007	0.012
0.061	0.015	0.023	-0.002	0.021	0.041	0.007	0.012	0.089
0.048	0.018	0.025	0.029	0.051	0.014	0.036	0.019	0.153
-0.024	-0.025	-0.048	-0.011	-0.009	-0.010	-0.013	-0.053	0.193

A-CH-38	A-CH-39	A-CH-40	A-CH-41	A-CH-42	A-CH-43	A-CH-44	A-CH-45	A-CH-46
-0.080	-0.122	0.065	0.321	0.400	-0.221	-0.211	-0.264	-0.150
-0.045	-0.011	0.303	0.477	0.034	0.269	0.101	0.098	-0.151
0.131	0.145	0.077	-0.073	0.139	0.104	0.372	0.254	0.029
0.110	0.182	-0.048	-0.042	0.173	0.025	-0.026	-0.156	0.028
-0.199	-0.085	-0.218	-0.312	-0.284	-0.099	-0.144	0.157	0.409
-0.744	-0.253	-0.169	0.097	-0.066	-0.057	0.041	0.158	-0.303
-0.020	0.008	0.283	0.059	-0.025	-0.213	-0.349	0.081	0.061
-0.025	-0.034	0.020	0.076	0.042	0.162	0.093	0.128	0.013
-0.147	-0.045	-0.061	0.518	-0.116	-0.229	-0.045	-0.118	-0.140
-0.003	0.000	0.197	0.279	0.174	0.169	0.049	-0.078	-0.045
-0.225	-0.189	0.127	0.162	0.056	0.061	0.010	-0.162	-0.151
-0.110	0.147	0.132	-0.092	0.055	0.095	-0.054	-0.023	0.087
-0.007	-0.103	-0.042	0.286	0.090	0.191	0.464	0.145	0.019
-0.032	-0.590	-0.613	-0.109	-0.277	0.058	0.596	0.182	-0.268
0.334	-0.062	-0.429	-0.019	-0.180	0.010	-0.167	-0.120	-0.094
-0.761	-0.131	-0.234	-0.028	0.082	0.204	-0.202	-0.053	-0.192
-0.103	0.008	-0.392	-0.161	-0.158	-0.628	-0.729	-0.249	0.086
0.312	0.174	0.202	0.409	1.293	0.274	0.390	0.122	0.229
0.065	0.144	0.150	0.255	0.243	0.513	0.381	0.344	0.219
0.148	-0.103	0.089	-0.032	0.181	-0.085	-0.174	0.047	0.189
0.555	0.095	-0.043	0.200	0.104	-0.112	-0.063	0.247	0.133
-0.364	-0.393	-0.058	0.162	0.347	-0.140	-0.050	-0.112	-0.291
-0.073	-0.065	-0.033	-0.055	-0.016	-0.029	0.051	-0.041	-0.084
-0.003	0.013	0.053	0.040	0.038	0.027	0.012	0.037	0.026
0.046	0.037	0.021	0.039	0.040	0.052	0.049	0.012	0.074
0.046	0.043	0.037	0.024	-0.069	0.015	0.049	0.048	-0.011
0.083	0.031	-0.044	-0.065	0.119	0.067	0.016	0.007	0.007
-0.059	-0.072	-0.058	-0.076	-0.059	-0.014	0.007	-0.041	-0.071
-0.060	-0.056	0.000	0.021	-0.075	0.043	0.070	0.052	-0.055
0.014	-0.047	-0.026	-0.023	0.040	-0.038	-0.045	-0.011	-0.012
-0.037	-0.094	-0.041	-0.057	-0.121	-0.076	-0.032	-0.121	-0.050
-0.060	-0.121	0.023	0.047	-0.045	-0.160	0.046	-0.027	-0.092
0.006	0.090	0.033	0.002	0.009	0.043	-0.018	-0.008	-0.012
0.078	0.059	0.039	-0.010	0.029	0.007	0.073	0.054	0.077
-0.054	-0.016	-0.001	-0.026	-0.069	-0.015	0.028	-0.031	-0.028
-0.056	0.041	0.021	0.014	-0.001	0.041	-0.036	0.044	0.100
0.016	0.026	0.038	0.022	0.035	0.030	0.044	0.007	0.022
0.037	0.032	0.010	0.004	0.024	0.019	0.052	0.055	0.019
0.022	0.010	-0.003	-0.052	-0.003	-0.050	-0.005	-0.007	-0.035

A-CH-47	A-CH-48	A-CH-49	A-CH-50	A-CH-51	A-CH-52
-0.112	-0.163	-0.213	0.010	0.157	-0.023
0.022	-0.038	0.035	0.084	0.455	0.283
0.106	0.200	0.169	0.090	0.186	0.092
-0.052	0.306	0.153	-0.021	0.145	-0.035
0.302	-0.287	0.012	-0.387	-0.526	-0.327
-0.310	-0.278	-0.359	-0.287	0.171	0.116
0.108	-0.043	0.041	0.158	0.087	0.274
0.009	0.208	0.071	0.026	0.074	0.228
-0.140	-0.119	0.058	-0.192	0.259	0.296
-0.099	0.014	0.019	-0.016	0.474	0.232
-0.136	-0.151	-0.179	0.057	0.159	0.064
-0.268	0.017	0.362	0.144	0.045	-0.074
-0.084	-0.090	-0.066	-0.072	0.505	0.211
-0.057	-0.035	-0.350	-0.920	0.131	0.068
-0.317	0.124	-0.317	-0.254	0.029	0.096
-0.136	-0.107	-0.600	-0.232	-0.130	0.093
-0.018	-0.050	-0.007	-0.174	-0.949	-0.628
0.076	0.212	0.403	0.064	-0.029	1.094
0.292	0.129	0.164	0.061	-0.034	0.303
0.122	0.021	0.142	0.133	0.036	0.031
0.095	0.216	0.165	0.022	0.057	0.083
-0.457	-0.547	-0.555	-0.138	0.034	0.264
-0.076	-0.099	-0.077	-0.041	0.006	-0.006
-0.009	0.008	0.032	0.005	0.040	0.054
0.049	0.083	0.089	0.034	0.045	0.049
0.012	0.019	0.034	0.075	0.152	0.055
-0.001	-0.033	0.067	0.023	0.036	0.029
-0.044	-0.047	-0.093	-0.081	-0.009	-0.073
-0.025	-0.066	-0.036	-0.039	0.051	0.010
-0.011	-0.019	-0.042	-0.040	-0.049	-0.014
-0.020	0.005	-0.075	-0.034	-0.014	-0.037
-0.156	-0.241	-0.075	-0.052	0.193	0.081
0.029	-0.035	0.055	0.033	-0.029	0.005
0.039	0.075	0.075	0.045	0.001	-0.005
-0.020	-0.029	-0.038	0.034	0.034	-0.027
-0.112	-0.135	-0.029	0.036	0.051	0.014
0.018	-0.004	0.017	0.057	0.047	0.033
0.013	0.031	0.018	0.013	0.009	0.009
-0.032	0.035	-0.005	-0.024	0.004	0.017
