# Supplementary material for: Transcutaneous electrical acupoint stimulation for children with attention-deficit/hyperactivity disorder: a randomized clinical trial
Source: Transl Psychiatry. 2022 Apr 21;12:165. doi: 10.1038/s41398-022-01914-0 (PMC9022403; doi:10.1038/s41398-022-01914-0)
Supplement: Supplementary file 4 — Supplement 4 [file 41398_2022_1914_MOESM4_ESM.pdf]

Channels	Interaction term (Bonferroni		Baseline and Week 4				TEAS and Sham TEAS			
			TEAS (Bonferroni		Sham TEAS (Bonferroni		Baseline (Bonferroni		Week 4 (Bonferroni	
1	0.984	(1.000)	0.956	(1.000)	0.934	(1.000)	0.777	(1.000)	0.797	(1.000)
2	0.627	(1.000)	0.827	(1.000)	0.639	(1.000)	0.237	(1.000)	0.562	(1.000)
3	0.799	(1.000)	0.946	(1.000)	0.771	(1.000)	0.966	(1.000)	0.733	(1.000)
4	0.550	(1.000)	0.804	(1.000)	0.550	(1.000)	0.306	(1.000)	0.823	(1.000)
5	0.920	(1.000)	0.923	(1.000)	0.811	(1.000)	0.603	(1.000)	0.707	(1.000)
6	0.987	(1.000)	0.890	(1.000)	0.908	(1.000)	0.811	(1.000)	0.796	(1.000)
7	0.740	(1.000)	0.727	(1.000)	0.905	(1.000)	0.711	(1.000)	0.440	(1.000)
8	0.933	(1.000)	0.856	(1.000)	0.765	(1.000)	0.914	(1.000)	0.990	(1.000)
9	0.965	(1.000)	0.947	(1.000)	0.898	(1.000)	0.084	(1.000)	0.094	(1.000)
10	0.980	(1.000)	0.970	(1.000)	0.998	(1.000)	0.250	(1.000)	0.237	(1.000)
11	0.888	(1.000)	0.941	(1.000)	0.900	(1.000)	0.894	(1.000)	0.940	(1.000)
12	0.749	(1.000)	0.842	(1.000)	0.799	(1.000)	0.032	(1.000)	0.032	(1.000)
13	0.905	(1.000)	0.964	(1.000)	0.830	(1.000)	0.761	(1.000)	0.888	(1.000)
14	0.033	(1.000)	0.848	(1.000)	0.951	(1.000)	0.489	(1.000)	0.615	(1.000)
15	0.943	(1.000)	0.899	(1.000)	0.820	(1.000)	0.670	(1.000)	0.731	(1.000)
16	0.764	(1.000)	0.877	(1.000)	0.787	(1.000)	0.310	(1.000)	0.506	(1.000)
17	0.978	(1.000)	0.881	(1.000)	0.851	(1.000)	0.550	(1.000)	0.528	(1.000)
18	0.899	(1.000)	0.606	(1.000)	0.736	(1.000)	0.528	(1.000)	0.442	(1.000)
19	0.429	(1.000)	0.602	(1.000)	0.550	(1.000)	0.072	(1.000)	0.451	(1.000)
20	0.815	(1.000)	0.837	(1.000)	0.901	(1.000)	0.055	(1.000)	0.028	(1.000)
21	0.702	(1.000)	0.834	(1.000)	0.740	(1.000)	0.292	(1.000)	0.117	(1.000)
22	0.898	(1.000)	0.795	(1.000)	0.937	(1.000)	0.538	(1.000)	0.435	(1.000)
23	0.661	(1.000)	0.661	(1.000)	0.516	(1.000)	0.555	(1.000)	0.997	(1.000)
24	0.807	(1.000)	0.829	(1.000)	0.575	(1.000)	0.207	(1.000)	0.326	(1.000)
25	0.952	(1.000)	0.720	(1.000)	0.657	(1.000)	0.999	(1.000)	0.949	(1.000)
26	0.915	(1.000)	0.946	(1.000)	0.934	(1.000)	0.062	(1.000)	0.080	(1.000)
27	0.733	(1.000)	0.971	(1.000)	0.655	(1.000)	0.611	(1.000)	0.342	(1.000)
28	0.994	(1.000)	0.834	(1.000)	0.826	(1.000)	0.765	(1.000)	0.772	(1.000)
29	0.627	(1.000)	0.987	(1.000)	0.482	(1.000)	0.024	(1.000)	0.098	(1.000)
30	0.634	(1.000)	0.841	(1.000)	0.636	(1.000)	0.219	(1.000)	0.506	(1.000)
31	0.866	(1.000)	0.905	(1.000)	0.906	(1.000)	0.130	(1.000)	0.080	(1.000)
32	0.917	(1.000)	0.835	(1.000)	0.951	(1.000)	0.422	(1.000)	0.349	(1.000)
33	0.985	(1.000)	0.985	(1.000)	0.945	(1.000)	0.518	(1.000)	0.501	(1.000)
34	0.746	(1.000)	0.928	(1.000)	0.713	(1.000)	0.397	(1.000)	0.669	(1.000)
35	0.832	(1.000)	0.862	(1.000)	0.635	(1.000)	0.767	(1.000)	0.976	(1.000)
36	0.706	(1.000)	0.887	(1.000)	0.696	(1.000)	0.705	(1.000)	0.917	(1.000)
37	0.001	(0.043)	＜0.001	(＜ 0.001）	0.812	(1.000)	0.881	(1.000)	＜0.001	(0.003)
38	0.607	(1.000)	0.926	(1.000)	0.413	(1.000)	0.407	(1.000)	0.120	(1.000)
39	0.850	(1.000)	0.916	(1.000)	0.710	(1.000)	0.100	(1.000)	0.100	(1.000)
40	0.676	(1.000)	0.928	(1.000)	0.496	(1.000)	0.916	(1.000)	0.683	(1.000)
41	0.965	(1.000)	0.907	(1.000)	0.859	(1.000)	0.104	(1.000)	0.104	(1.000)
42	0.931	(1.000)	0.852	(1.000)	0.948	(1.000)	0.304	(1.000)	0.248	(1.000)
43	0.921	(1.000)	0.999	(1.000)	0.890	(1.000)	0.536	(1.000)	0.447	(1.000)
44	0.778	(1.000)	0.973	(1.000)	0.666	(1.000)	0.626	(1.000)	0.908	(1.000)
45	0.748	(1.000)	0.994	(1.000)	0.644	(1.000)	0.005	(0.283)	0.016	(0.815)
46	0.918	(1.000)	0.992	(1.000)	0.892	(1.000)	0.587	(1.000)	0.668	(1.000)
47	0.591	(1.000)	0.900	(1.000)	0.527	(1.000)	0.842	(1.000)	0.583	(1.000)

**48**	**0.532**	**(1.000)**	**0.992**	**(1.000)**	**0.371**	**(1.000)**	**0.410**	**(1.000)**	**0.101**	**(1.000)**
**49**	**0.006**	**(0.324)**	**0.896**	**(1.000)**	**0.809**	**(1.000)**	**0.153**	**(1.000)**	**0.184**	**(1.000)**
**50**	**0.929**	**(1.000)**	**0.956**	**(1.000)**	**0.943**	**(1.000)**	**0.350**	**(1.000)**	**0.420**	**(1.000)**
**51**	**0.854**	**(1.000)**	**0.885**	**(1.000)**	**0.685**	**(1.000)**	**0.798**	**(1.000)**	**0.970**	**(1.000)**
**52**	**0.842**	**(1.000)**	**0.972**	**(1.000)**	**0.805**	**(1.000)**	**0.805**	**(1.000)**	**0.007**	**(0.361)**
